# Androgen receptor and chemokine receptors 4 and 7 form a signaling axis to regulate CXCL12-dependent cellular motility

**DOI:** 10.1186/s12885-015-1201-5

**Published:** 2015-03-31

**Authors:** Jordy J Hsiao, Brandon H Ng, Melinda M Smits, Jiahui Wang, Rohini J Jasavala, Harryl D Martinez, Jinhee Lee, Jhullian J Alston, Hiroaki Misonou, James S Trimmer, Michael E Wright

**Affiliations:** 1 Department of Molecular Physiology & Biophysics, The University of Iowa, Carver College of Medicine, 51 Newton Road, Iowa City, Iowa 52242 USA; 2 Department of Pharmacology, Davis Genome Center, University of California Davis School of Medicine, One Shields Avenue, Davis, California 95616 USA; 3 Graduate School of Brain Science, Doshisha University, Kyoto, Japan; 4 Department of Neurobiology, Physiology and Behavior and Department of Physiology and Membrane Biology, University of California Davis, School of Medicine, One Shields Avenue, Davis, California 95616 USA

**Keywords:** Androgen receptor, CXCR4, CXCR7, Cell motility, Prostate cancer

## Abstract

**Background:**

Identifying cellular signaling pathways that become corrupted in the presence of androgens that increase the metastatic potential of organ-confined tumor cells is critical to devising strategies capable of attenuating the metastatic progression of hormone-naïve, organ-confined tumors. In localized prostate cancers, gene fusions that place ETS-family transcription factors under the control of androgens drive gene expression programs that increase the invasiveness of organ-confined tumor cells. C-X-C chemokine receptor type 4 (CXCR4) is a downstream target of ERG, whose upregulation in prostate-tumor cells contributes to their migration from the prostate gland. Recent evidence suggests that CXCR4-mediated proliferation and metastasis of tumor cells is regulated by CXCR7 through its scavenging of chemokine CXCL12. However, the role of androgens in regulating CXCR4-mediated motility with respect to CXCR7 function in prostate-cancer cells remains unclear.

**Methods:**

Immunocytochemistry, western blot, and affinity-purification analyses were used to study how androgens influenced the expression, subcellular localization, and function of CXCR7, CXCR4, and androgen receptor (AR) in LNCaP prostate-tumor cells. Moreover, luciferase assays and quantitative polymerase chain reaction (qPCR) were used to study how chemokines CXCL11 and CXCL12 regulate androgen-regulated genes (*ARG*s) in LNCaP prostate-tumor cells. Lastly, cell motility assays were carried out to determine how androgens influenced CXCR4-dependent motility through CXCL12.

**Results:**

Here we show that, in the LNCaP prostate-tumor cell line, androgens coordinate the expression of CXCR4 and CXCR7, thereby promoting CXCL12/CXCR4-mediated cell motility. RNA interference experiments revealed functional interactions between AR and CXCR7 in these cells. Co-localization and affinity-purification experiments support a physical interaction between AR and CXCR7 in LNCaP cells. Unexpectedly, CXCR7 resided in the nuclear compartment and modulated AR-mediated transcription. Moreover, androgen-mediated cell motility correlated positively with the co-localization of CXCR4 and CXCR7 receptors, suggesting that cell migration may be linked to functional CXCR4/CXCR7 heterodimers. Lastly, CXCL12-mediated cell motility was CXCR7-dependent, with CXCR7 expression required for optimal expression of CXCR4 protein.

**Conclusions:**

Overall, our results suggest that inhibition of CXCR7 function might decrease the metastatic potential of organ-confined prostate cancers.

**Electronic supplementary material:**

The online version of this article (doi:10.1186/s12885-015-1201-5) contains supplementary material, which is available to authorized users.

## Background

Prostate cancer is among the most common and deadly of cancers that afflict men in the United States, and is second only to lung cancer with respect to cancer-related death [1]. Organ-confined prostate cancer is readily cured through radical prostatectomy and has a 5-year relative survival rate of nearly 100% [1]. Notably, in the case of metastatic prostate cancer, the survival rate is only ~29% [1]. Given that current therapies are ineffective at curing these more advanced cancers, it has become common to treat patients at the organ-confined stage of disease. However, this results in the significant overtreatment of low-risk, organ-confined prostate cancer, as the majority of the early-stage tumors are indolent [2]. Identifying biomarkers linked to the metastasis of prostate tumor cells will be critical to distinguish tumors with a high risk of progression from those that are truly indolent.

Approximately 50% of organ-confined prostate cancers harbor chromosomal rearrangements that lead to gene fusions involving the transcription factor-encoding genes of the ETS family (*e.g.,* ERG, ETV1) [3]. This places them under the control of androgen-regulated gene promoters such as TMPRSS2, so that their expression is upregulated in the presence of androgens [3]. In tumor cells harboring *PTEN* loss-of-function mutations, androgens acting through TMPRSS2-ETS gene fusions promote prostate tumorigenesis by upregulating ETS-responsive target genes that promote cell motility, cell proliferation, and androgen metabolism [4-7], thereby increasing the metastatic potential of the cells [5,6]. Thus, the products of such genes in low-grade, organ-confined prostate cancers might represent novel biomarkers of significant disease.

Transcriptional upregulation of the chemokine receptor 4 gene (*CXCR4*) in organ-confined tumor cells that overexpress the ETS-related gene ERG (*i.e.*, TMPRSS2-ERG fusion) increases the motility of prostate tumor cells *in vitro* [8]. CXCR4 is a seven-transmembrane G protein-coupled receptor involved in the development, migration, and morphogenesis of cells in the hematopoietic, cardiovascular, and central nervous systems [9-11]. It plays an important role in the homing of hematopoietic stem cells [12], particularly to bone marrow [13-15], which is the most frequent site of metastasis for prostate cancers [14].

CXCR4 forms a signaling axis with chemokine ligand 12 (CXCL12) and chemokine receptor 7 (CXCR7) [16]. CXCL12 binds both CXCR4 and CXCR7, inducing Gαi-dependent signaling through CXCR4 and Gαi-independent signaling through CXCR7 [17-19]. CXCL12 mediates the homing of cells that express CXCR4 [13], and high levels of CXCL12 are associated with the preferential metastasis of prostate-cancer cells to the bone [14,20-24]. *In vitro* studies have recently shown that androgens regulate the expression of CXCR4 to increase the metastatic potential of prostate-tumor cells [8,25].

Androgens stimulate CXCR4 expression through two pathways: 1) in TMPRS22-ERG positive cells they promote the transcriptional actions of ERG [8], and 2) in TMPRS22-ERG negative cells they work through the transcription factor Krüppel-like factor 5 (KLF5) [25]. In contrast, androgens influence expression of the CXCR7 mRNA in a manner dependent upon cell malignancy; they promote CXCR7 expression in immortalized, non-malignant human prostate epithelial cells (*e.g.,* HPr-1AR) [26], but repress it in neoplastic prostate epithelial cells (*e.g.,* LNCaP) [27,28]. Notably, in clinical prostate samples, androgenic control of the expression of CXCR4 and CXCR7 is regulated in reciprocal fashion. For example, analysis of the Oncomine database showed that expression of the CXCR4 mRNA in normal prostate epithelial cells is lower than that in organ-confined neoplastic counterparts (Table 1) [29,30]. This suggests that in hormone-naïve patients with organ-confined prostate tumors with presumably normal circulating levels of androgens (*e.g.,* ~10-34 nM testosterone) [31], expression of the CXCR4 mRNA becomes de-repressed. Conversely, expression of the CXCR7 mRNA is reduced in organ-confined prostate cancer cells relative to normal prostate epithelial cells. This finding suggests that in patients with hormone-naïve, organ-confined prostate-cancer cells, expression of the CXCR7 mRNA is repressed or deactivated [32-35].

**Table 1:** **Gene expression profiles of CXCR7, CXCR4, CXCL11, CXCL12 in human prostate cancer samples**

Gene name	Cancer vs. normal	References
*CXCR7*	^↓^ [32-35]	Welsh *JB et*., [32]
		La Tulippe *E et al.,* [33]
		Luo JH *et al*., [34]
		Liu P *et al*., [35]
*CXCR4*	↑ [30,32]	Yu YP *et al.,* [29]
		Wallace *et al.,* [30]
*CXCL11*	↑ [32]	Welsh JB *et al*., [32]
*CXCL12*	↑ [34]	Luo JH *et al.,* [34]

Legend: ↑indicates increased expression.

^↓^indicates decreased expression.

p-value <0.05, 2-fold change.

In summary, androgens appear to repress transcription of the CXCR4 mRNA and to stimulate that of the CXCR7 mRNA in normal prostate epithelial cells, but to have the opposite effect in the neoplastic prostate epithelial cells of organ-confined cancers. In this study we detail how the synthetic androgen R1881 regulates the CXCR4/CXCR7 axis to control CXCL12-mediated motility of LNCaP prostate tumor cells. Physical and functional interactions were detected between AR and CXCR7 in cells to demonstrate the biochemical integration of androgen signaling and cellular motility machinery at the molecular level in LNCaP prostate tumor cells. Furthermore, our findings demonstrate that CXCR7 is a critical determinant of motility in response to CXCL12, and that it acts by upregulating CXCR4 protein levels in these cells.

## Methods

### Reagents

The following reagents were purchased from the indicated vendors: AR agonist R1881 (methyltrienolone) (Perkin Elmer Life Sciences, Waltham, MA); CXCL11 (672-IT) and CXCL12 (2716-SD) ligands (R&D Systems, Minneapolis, MN); double-stranded experimentally validated siRNAs for scrambled control (1027281), AR (SI02757258), CXCR4 (SI02664235), CXCR7 (SI02660644) (Qiagen, Valencia, CA), and CXCR7 (109229) (Life Technologies, Chicago, IL); RNeasy Mini kit, RT^2^ qPCR primers for AR (PPH01016A), CXCR7 (PPH01182F), CXCR4 (PPH00621A), PSA (PPH01002B), FASN (PPH01012B), NKX3.1 (PPH02267C), TMPRSS2 (PPH02262C) (Qiagen); Oligofectamine Transfection Reagent, 4%-12% SDS-polyacrylamide gels, Superscript III enzyme, CyQUANT Cell Proliferation Assay Kit (Life Technologies); iQ SYBR-Green Supermix, Precision Plus Prestained Protein Standards, goat anti-mouse horseradish peroxidase (HRP)-conjugated secondary antibody, goat anti-rabbit HRP-conjugated secondary antibody (BioRad, Hercules, CA); mouse monoclonal AR antibody (AR441), rabbit polyclonal AR antibody (N-20), mouse monoclonal SBP antibody (SB19-C4) (Santa Cruz Biotechnology, Santa Cruz, CA); rabbit polyclonal CXCR7 antibodies (ab38089 [a.a. 1–100], and ab72100 [a.a. 106–117, QHNQWPMGELTC]), rabbit polyclonal CXCR4 antibody (ab2074) (Abcam, Cambridge, MA); rabbit polyclonal CXCR4 antibody (PAB9849) (Abnova, Taipei, Taiwan); mouse monoclonal GM130 antibody (BD Transduction Laboratories, San Jose, CA); rabbit polyclonal PSA antibody (DAKO, Carpinteria, CA); mouse monoclonal PSMA antibody (Meridian Life Science Inc, Memphis, TN); rabbit polyclonal Histone H3 antibody, rabbit monoclonal GAPDH antibody (14C10) (Cell Signaling Technology, Beverly, MA); BCA Protein Assay Kit and ECL Western Blotting Substrate Kit (ThermoFisher Scientific, Waltham, MA); Hyperfilm ECL film (GE Healthcare, Piscataway, NJ); fetal bovine serum, charcoal-stripped fetal bovine serum (Hyclone Laboratories, Logan, UT); GeneRuler 1 kb DNA Ladder (MBI Fermentas, Hanover, MD); Protein Deglycosylation Mix (P6039S, New England BioLabs, Ipswich, MA); Synthetic peptides to CXCR7 (a.a. 348–362, RVSETEYSALEQSTK) and AR (a.a. 299–315, KSTEDTAEYSPFKGGY) were synthesized by Alpha Diagonistics (San Antonio, TX).

### Generation of the polyclonal rabbit pAbCXCR7 antibody to CXCR7

The pAbCXCR7 polyclonal antibody was generated, by Alpha Diagnostics, against a synthetic peptide encoded by NCBI’s original CXCR7 cDNA sequence, which contained asparagine and alanine residues at amino acid positions 360 and 361 (348-RVSETEYSALEQ*NA*K-362). Updated NCBI cDNA sequences for CXCR7 are polymorphic at codons S360N and T361A; therefore, the original antibody was subjected to peptide affinity-purification. A C-terminal polypeptide containing serine and threonine residues at these respective positions was used to enrich for antibodies that are cross-reactive to this CXCR7 isoform (348-RVSETEYSALEQ*ST*K-362) based upon the peptide affinity antibody purification protocol [36]. The 15 amino acid residues of human CXCR7 (348-RVSETEYSALEQ*ST*K-362) were subjected to a BLAST analysis to the UniProt Human protein sequence database to identify high scoring polypeptide matches. The affinity-purified antibody (pAbCXCR7) was used throughout the experiments described here.

### Cell lines

LNCaP, 22Rv1, DU145, and PC3 cells were obtained from the American Tissue Type Culture Collection. LNCaP and 22Rv1 cells were grown in phenol red-deficient RPMI 1640 medium (Invitrogen) containing either 10% fetal bovine serum (FBS) or 10% charcoal/dextran-treated (CS-FBS). DU145 and PC3 cells were grown in phenol red-deficient high-glucose Dulbecco’s modified Eagle’s medium (DMEM) containing 10% FBS. All cell lines were supplemented with penicillin/streptomycin/glutamine and maintained at 37°C and 5% CO_2_.

For the generation of the SBP and C7-SBP LNCaP cell lines, the mammalian expression vector pCMV-SPORT6-CXCR7 was used as a template for PCR-based amplification of CXCR7, which was subcloned into the synthetic pcDNA3-streptavidin binding peptide (SBP)-FLAG expression vector (Genscript, Piscataway, NJ). Amplification of CXCR7 was carried out using the Advantage GC-2 polymerase (Clontech, Mountain View, CA), and the cDNA was cloned in-frame into the C-terminus of the 5′ EcoRI and 3′ XhoI restriction sites of the pcDNA3-SBP-FLAG vector. The SBP sequence used was 5′-ATGGACTACAAGGACGACGAC-3′. Oligonucleotide primers (Integrated DNA Technologies, Coralville, IA) used for cloning CXCR7-SBP were: the 5′ EcoRI primer, 5′-GATCGAATTCGCCACCATGGATCTGCATCTCTTCGACTACTCAGAGCCAGGGAAC-3′, and the 3′ XhoI primer, 5′-GATCCTCGAGTTTGGTGCTCTGCTCCAAGGCAGAGTACTC-3′. Individual pcDNA3-SBP-FLAG and pcDNA3-CXCR7-SBP-FLAG cDNAs were transfected into LNCaP cells and stable clones were selected under G418 selection.

### Competition experiments

All peptide/antibody blocking experiments were performed by pre-incubating the pAbCXCR7 antibody with 10 μg of AR (a.a. 299–315) or CXCR7 peptide (a.a. 348–362) for 1 hr at 37**°**C in Tris-buffered saline containing 0.1% Tween 20 (TBST) and 5% bovine serum albumin (BSA). These antibody/peptide mixtures were used for western blot analyses as detailed in the immunoblotting section.

### Western blot

Whole cell lysates (*WCL*) were derived from *AD-* and *AS*-LNCaP cells solubilized in 0.3 ml of buffer A (50 mM Tris–HCl, 150 mM NaCl, 5 mM EDTA, pH 7.4, 1% SDS) and quantified via the BCA Assay. 4 μg of microsomal protein were heated to 95°C for 5 mins and subjected to western blot analysis.

For the western blot analysis of neoplastic epithelial cell lines (Additional file 1: Figure S1.C), total proteins were resolved into a 12% SDS polyacrylamide gel, and the molecular weight marker used was different compared to other western blot experiments.

For knockdown experiments, 96 hr siRNA-transfected LNCaP cells were solubilized in 0.3 ml of buffer A and heated to 95°C for 5 min. Total protein in each lysate was quantified using the Pierce BCA Protein Assay Kit, and 4 μg each were subjected to SDS-PAGE (4%-12% gradient precast gels). Proteins were transferred onto a PVDF membrane, incubated in TBST, and blocked with 5% nonfat milk (w/v) for 1 hr. Membranes were incubated overnight at 4°C in TBST containing 5% BSA and one of the following antibodies: 1:500 dilution of CXCR4 rabbit polyclonal antibody; 1:5000 dilution of rabbit polyclonal CXCR7 antibody (pAbCXCR7); 1:1000 dilution of rabbit polyclonal AR (N-20) antibody; 1:250 dilution of mouse monoclonal AR (441) antibody; 1:1000 dilution of rabbit polyclonal PSA antibody; 1:000 dilution of mouse monoclonal H3 antibody; 1:1000 dilution of mouse monoclonal GM130 antibody. Membranes underwent three 5 min TBST washes before a 1 hour, room temperature incubation with secondary antibody (*i.e.*, goat anti-mouse or goat anti-rabbit horseradish peroxidase secondary) at a 1:10,000 dilution in TBST containing 5% BSA. Three more 5 min TBST washes were performed, and immunoreactive bands were developed and visualized using ECL Western Blotting Substrate. The blots were exposed to Hyperfilm ECL film for < 5 min. 4 μg of total protein lysates were resolved on SDS-PAGE and total protein was visualized by silver staining.

### qPCR analysis

Total RNA was extracted using the RNeasy Mini kit following the manufacturer’s protocol. RNA (0.5 μg) was converted to cDNA with Superscript III enzyme, and qPCR was performed with iQ SYBR-Green Supermix using *AR*, *CXCR7*, *CXCR4*, *FASN*, *NKX3.1*, *TMPRSS2*, and *PSA* primers in a CFX Connect real-time PCR thermocycler (BioRad).

### Subcellular fractionation

Subcellular fractionation was carried out on LNCaP cells grown in 10% FBS for 96 hr or 10% CS-FBS for 72 hrs and then treated with either vehicle (ethanol, *AD*) or androgen (1nM R1881, *AS*) for 24 hrs, using the Subcellular Protein Fractionation Kit for Cultured Cells (Thermo Scientific), according to the manufacturer’s guidelines.

For acute CXCR7 ligand-treatment experiments, the androgen-depleted LNCaP cells were treated with vehicle, 100 nM CXCL11, or 100 nM CXCL12 for 30 min, lysed, and subjected to subcellular fractionation through differential centrifugation. Briefly, harvested cells were incubated in hypotonic solution (10 mM Hepes, 1.5 mM MgCl_2_, 10 mM KCl, pH 7.9) for 10 min and passed through an 18-gauge syringe 15 times. Nuclei were pelleted via centrifugation at 600 × g for 20 min at 4°C and then resuspended in nuclear extraction buffer (20 mM Hepes, 600 mM KCl, 25% glycerol, 1.5 mM MgCl_2_, 0.2 mM ZnCl_2_, pH 7.9). The supernatant was decanted and subjected to ultra-centrifugation at 100,000 × g for 3 hrs at 4°C to separate the membranes (*i.e.*, crude microsomes) from the cytosol.

### Structured illumination/ApoTome microscopy

*AD-* and *AS-*LNCaP cells were fixed and permeabilized with freshly depolymerized 4% formaldehyde, 0.1% TX-100, in PBS at 4°C for 30 min., washed, and blocked in Blotto (3% nonfat dry milk powder, 0.1% TX-100 in TBS). Cells were simultaneously stained with rabbit anti-CXCR7 polyclonal antibody and mouse anti-EEA1 monoclonal antibody (BD Biosciences) for 1 hr at room temperature. These were then stained with Alexa 488 conjugated goat anti-rabbit IgG, Alexa 594 goat anti-mouse IgG, and DAPI nuclear dye (Invitrogen) for 1 hr at room temperature. Cells were mounted in Prolong Gold, and immunofluorescence was imaged on a Zeiss Axiovert 200 microscope equipped with an Apotome structured illumination system under a 63X/1.4 NA objective. Optical Z-sections (24–32 Z-sections, 0.4 mm thick) were acquired from each sample and a cross-sectional view was generated using Axiovision software (in “Cut View” processing mode). Reconstruction of the entire Z-stack from individual optical sections was performed using Extended Focus processing.

### Immunofluorescence

For CXCL11 and CXCL12 ligand treatment experiments, LNCaP cells depleted of androgen for 72 hrs were treated with BSA (0.1%), CXCL11 (100 nM), or CXCL12 (100 nM) for 30 min. Media was removed, and cells were fixed in DPBS containing 4% formaldehyde for 20 min at room temperature. After three washes with DBPS, cells were blocked in Blotto and then processed for immunofluorescence imaging by staining the cells with CXCR7, CXCR4, or AR antibodies. DNA was labeled with DAPI, F-actin was labeled with Texas Red-X phalloidin, and samples were labeled with Alexa Fluor 488 goat anti-rabbit or Alexa Fluor 488 goat anti-mouse secondary antibodies.

For the semi-permeabilization of cells treated with different androgen doses, cells were fixed in DPBS containing 4% formaldehyde and 4% methanol for 20 min at room temperature. After three washes with DBPS, cells were blocked in Blotto and then processed for immunofluorescence imaging by staining the cells with CXCR7, CXCR4, or AR antibodies. DNA was labeled with DAPI, F-actin was labeled with Texas Red-X phalloidin, and samples were labeled with Alexa Fluor 488 goat anti-rabbit or Alexa Fluor 488 goat anti-mouse secondary antibodies.

### Transfection of siRNAs

LNCaP cells were seeded at 2,000 cells/cm^2^ in antibiotic-deficient medium A for 24 hrs prior to transfection. Experimentally validated control, AR, CXCR7, and CXCR4 siRNAs were transfected into the cells for 72 hrs at a final concentration of 100 nM using Oligofectamine Reagent (Invitrogen) according to the manufacturer’s guidelines. Light micrographs were taken using the VWR™ VistaVision™ inverted microscope at 10× magnification.

### Streptavidin affinity purification of SBP-tagged CXCR7

SBP and C7-SBP cells were each grown in one plate of 500 cm^2^ cell culture dish (Corning Inc., Corning, NY) to 80% confluency for 96 hr. Cells were collected with DPBS, and subjected to hypotonic lysis for subcellular fractionation. The collected cell pellets were resuspended in 5 ml of hypotonic buffer (10 mM HEPES, 1.5 mM MgCl_2_, 10 mM KCl, pH 7.9 with 10 mM DTT and 1× protease inhibitor cocktail [PIC]), and incubated on ice for 10 min. The cells were then subjected to nitrogen cavitation at 100 psi for 5 min, and the nuclei were pelleted by centrifugation at 10,000 × g for 20 min at 4°C. The supernatant was then subjected to ultra-centrifugation at 100,000 × g for 3 hr at 4°C, to separate the membranes (crude microsome) from the cytosol. The membrane proteins were extracted from the membrane pellet using 1% digitonin in microsome buffer (20 mM Tris, 150 mM NaCl, 0.1 mM CaCl_2_, 0.1 mM MnCl_2_, pH 7.5 with 10 mM DTT and 1 × PIC) and rotated end-over-end overnight at 4°C. Detergent-insoluble material was removed by centrifugation at 100,000 × g for 3 hr at 4°C. The isolated membrane proteins were analyzed by silver staining to determine protein concentration. 2 mg of SBP and C7-SBP membrane proteins were incubated with 50 μl of equilibrated Streptavidin Plus UltraLink Resin (Thermo Scientific) beads and rotated end-over-end overnight at 4°C. The flow-through was collected and the beads were washed three times (200 μl/wash) with microsome buffer containing 0.1% of digitonin, 10 mM DTT and 1 × PIC. The washes were pooled and Streptavidin bound proteins were eluted with a total of 200 μl of 5 mM D-Biotin in microsome buffer.

### Luciferase assay

LNCaP cells were seeded into Falcon (BD Biosciences) 48-well tissue culture dishes at a density of 30,000 cells/cm^2^ and incubated for 24 hrs in phenol red-deficient RPMI 1640 medium containing 10% CS-FBS. Cells in each well were transfected, in triplicate, with Lipofectamine 2000 (Invitrogen) and 335 ng of total plasmid DNA as follows: pGL4.10-Luc2-*probasin* [10 ng], pRLSV40 *Renilla* [25 ng] [Promega], increasing amounts (30 ng, 100 ng, 300 ng) of mammalian expression vector, and pcDNA3 (270 ng, 200 ng, 100 ng) to have a total of 335 ng of plasmid DNA. Vehicle (ethanol) or androgen (1 nM R1881) was added 24 hrs after transfection, and total cell lysates were assessed for luciferase activity 24 hrs later using the Dual-Luciferase Reporter (DLR) Assay System (Promega) according to the manufacturer’s detailed protocol. Values for firefly and *Renilla* luciferase were determined using the Veritas microplate luminometer (Turner Biosystems, Sunnyvale, CA). The means and standard deviations for all firefly luciferase values were calculated and statistical significance (*p ≤ 0.05, n = 3) between control and experimental transfected cells was determined with Student’s *t*-test for the androgen-treatment group.

For siRNA knockdown luciferase assays, LNCaP cells were seeded into Falcon (BD Biosciences) 48-well tissue culture dishes at a density of 30,000 cells /cm^2^. After 24 hrs in phenol red-deficient RPMI 1640 growth medium supplemented with 10% charcoal-stripped FBS, the cells were transfected with Lipofectamine 2000. Transfections were carried out in triplicate with pGL4.10-Luc2-*probasin* (10 ng) and pRLSV40 *Renilla* (25 ng) for 48 hrs and then treated with vehicle (ethanol) or androgen (1 nM R1881) for 24 hrs. The means and standard deviations for all firefly luciferase values were calculated, and the statistical significance (*p ≤ 0.05, n = 3) was determined between cells transfected with control or experimental siRNAs for each treatment group using the Student’s *t*-test.

For siRNA knockdown luciferase experiments with ligand treatment, either vehicle, 100 nM CXCL11, or 100 nM CXCL12 was added to the cells 48 hrs after transfection for 30 mins, and vehicle (ethanol) or androgen (1 nM R1881) was added for 12–18 hrs. Total cell lysates were assessed for luciferase activity using the Dual-Luciferase Reporter (DLR) Assay System (Promega) according to the manufacturer’s protocol. The means and standard deviations for all firefly luciferase values were calculated, and the statistical significance (*p ≤ 0.05, n = 3) was determined between cells transfected with control and experimental siRNAs for each treatment group using the Student’s *t*-test.

### Isolation of membrane and membrane-associated glycoproteins

Crude microsomes derived from lysates of *AD* and *AS* (*i.e.,* 0.1 nM, 1.0 nM, and 10 nM R1881) LNCaP cells were solubilized in 50 mM Tris–HCl, 150 mM NaCl, 0.1 mM CaCl_2_, 0.1 mM MnCl_2_, 1% Digitonin, pH 7.5, and quantified by BCA assay. 10 mg of protein from each condition were subjected to lectin-affinity chromatography, using wheat germ agglutinin (WGA) beads to isolate *N*-linked glycoproteins and concanavalin A (ConA) beads for *O*-linked glycoproteins. Glycoproteins were eluted with sugars according to the manufacturer’s guidelines. Samples were subjected to Western blot analysis as described below with the CXCR4 (1:1000), pAbCXCR7 (1:5000), PSA (1:1000) and PSMA (1:500) antibodies.

### Boyden-chamber cell motility assay

#### Androgen treatment

LNCaP cells grown in phenol red-deficient RPMI 1640 medium containing 10% FBS for 72 hrs were dissociated using Accutase (Invitrogen) and counted with a hemocytometer. 75,000 cells were seeded per well in 24-well plates with phenol red-deficient RPMI medium containing 1% charcoal-stripped FBS. The top and bottom of Biocoat control inserts (BD Biosciences, Palo Alto, CA), an 8 μm membrane pore size, were coated with 5 μg/ml of fibronectin in DPBS for 2 hrs at 37°C and subsequently washed with 1 DPBS and dried at 25°C. Cells were seeded into the top chamber, and the bottom chamber was filled with phenol red-deficient RPMI medium containing 1% charcoal-stripped FBS plus androgen (0, 0.1, 1, or 10 nM R1881). Migration was allowed to proceed at 37°C under 5% CO_2_ for 18–24 hrs. The cells were then fixed with −20°C methanol for 10 min at 25°C, and inserts were stained with 0.5% crystal violet (Sigma) in 25% methanol for 10 min at 25°C. Inserts were washed with ddH_2_O for 5 mins at 25°C and visualized under a light microscope to count cells. The means and standard deviations for counted cells were calculated, and *ANOVA* was used to determine statistical significance (*p ≤ 0.05, n = 3) between vehicle (ethanol) and androgen-treated cells.

#### CXCL12 treatment

Cell migration assays were prepared exactly as described for androgen treatment experiments, except cells were treated with 0.1% BSA or CXCL12 at 0.3, 3, or 30 nM in the presence of 1 nM R1881. The means and standard deviations for counted cells were calculated and *ANOVA* was used to determine statistical significance (*p ≤ 0.05, n = 3) between vehicle- (ethanol) and androgen-treated cells.

#### siRNA experiments

LNCaP cells were plated at a density of 3,000 cells/cm^2^ on 6-well tissue culture plates and incubated for 24 hrs at 37°C, in 2 ml of phenol red-deficient RPMI 1640 growth medium supplemented with 10% FBS. Cells were then transfected with control, AR, CXCR7, or CXCR4 siRNA at a final concentration of 100nM using the Oligofectamine Transfection Reagent according to the manufacturer’s guidelines. After 72 hrs, the cells were dissociated using Accutase and seeded into the top chamber of an insert as described above. The bottom chamber was filled with phenol red-deficient RPMI medium with 1% charcoal-stripped FBS containing 1 nM androgen (R1881) and treated with 0.1% BSA, 0.003, 0.03, or 0.3 nM CXCL12. Cell motility was measured and analyzed as detailed above. The means and standard deviations were determined, and *ANOVA* was used to determine statistical significance (*p ≤ 0.05, n = 3) between control and experimental transfected cells.

## Results

### Molecular characterization of a C-terminal polyclonal antibody to CXCR7

Androgens are known to induce CXCR4-dependent cell motility in prostate-cancer cells by upregulating CXCR4 [8,25]. CXCR7 is a key regulator of CXCR4-dependent motility [17,18,37-39], and we have previously shown that it is an androgen-sensitive microsomal protein in the LNCaP prostate-cancer cell line [40]. Therefore, we set out to examine how androgens regulate the subcellular localization of CXCR7 and to determine the role of this protein in CXCR4-mediated motility in prostate-cancer cells. Commercial CXCR7 antibodies are available but have not been subjected to careful molecular characterization in prostate-cancer cells. Therefore, we developed a polyclonal antibody (*i.e.*, pAbCXCR7) against the CXCR7 C-terminus (*i.e.*, residues 348–362) to use in exploring, in depth, the subcellular localization and expression of this protein in prostate-cancer cells.

**Figure 1:**
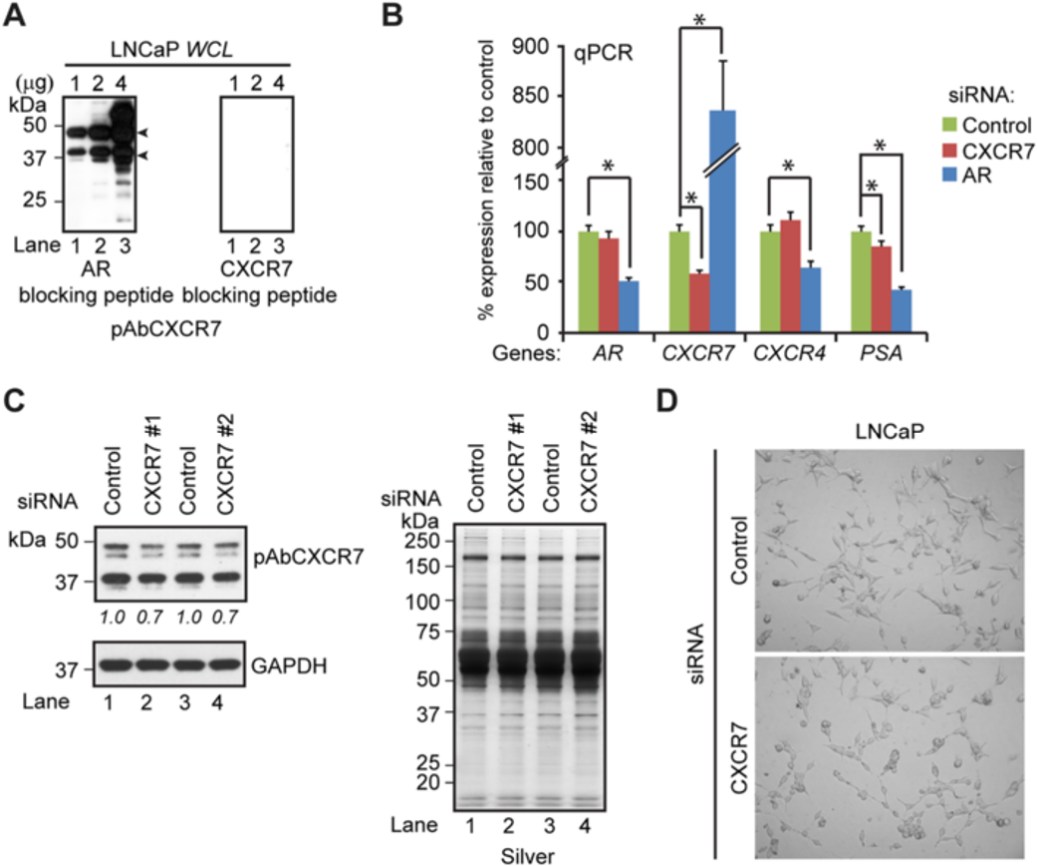
**CXCR7 expression in prostate-cancer cells. (A)** Western blot of 1, 2, and 4 μg of LNCaP total lysate with pAbCXCR7 antibody in the presence of the non-competitive AR peptide (a.a. 299–315, left panel) or the CXCR7 blocking peptide (a.a. 348–362, right panel) as detailed in Materials and Methods section. The CXCR7 bands are indicated by arrowheads. **(B)***AR*, *CXCR7*, *CXCR4*, and *PSA* gene expressions in LNCaP cells transfected with AR, CXCR7, or scrambled control siRNA. RNA was isolated 72 hrs post-transfection and measured by qPCR. Student’s *t*-test was used to calculate significant differences (*p ≤ 0.05, n = 3) between control and experimental cells. **(C)** Western blot (left panel) of whole cell lysates from LNCaP cells transfected with control or two experimentally-validated CXCR7 siRNAs (CXCR7 #1 or #2) for 72 hrs using antibodies to pAbCXCR7 and GAPDH. Silver-stained gel demonstrated equal protein loading across samples (right panel). The densitometry values were labeled below the blot and normalized to the control transfected cells loaded with the same amount of total proteins. **(D)** Light microscopy of LNCaP cells transfected with control or CXCR7 siRNA for 72 hrs.

Initial western blot characterization of the pAbCXCR7 revealed two prominent immunoreactive CXCR7 bands at approximately 40 kDa and 48 kDa (Figure 1A, left panel, lane 1). More importantly, pAbCXCR7 immunoreactivity was specific for CXCR7, as immunoreactive bands were competitively removed when pAbCXCR7 was pre-incubated with the C-terminal CXCR7 peptide (*i.e.*, amino acids 348–362), but not with the non-competitive AR peptide (*i.e.*, amino acids 299–315) (Figure 1A, right panel versus left panel).

To further demonstrate the specificity of pAbCXCR7 for the CXCR7 protein, LNCaP cells were transfected with two experimentally validated siRNAs directed against CXCR7. Western blot analysis showed a reduction in CXCR7 protein that was concordant with the ~30% reduction in CXCR7 mRNA determined by qPCR in CXCR7 knockdown cells relative to control knockdown cells (Figure 1B and C). Phenotypically, 72-hr CXCR7 knockdown cells were noticeably more rounded-up and loosely attached to the dish when compared to control knockdown cells (Figure 1D). Despite repeated siRNA experiments, a reduction in CXCR7 mRNA or protein beyond ~30% was unattainable in siRNA-transfected LNCaP cells. This outcome most likely reflects the finding that CXCR7 expression is required for cell viability in prostate-cancer cells [41]. Two independent, commercially available rabbit polyclonal CXCR7 antibodies (*i.e.*, Ab72100 and Ab38089) also confirmed that CXCR7 protein was reduced in CXCR7 knockdown cells relative to control cells (Additional file 1: Figure S1.A).

We further tested the specificity of the antibody by assessing its ability to detect epitope-tagged CXCR7 heterologously expressed in LNCaP cells. Western blot analyses were performed on two LNCaP-derived cell lines. The first was the C7-SBP cell line, which stably expresses a CXCR7 fusion protein that contains a C-terminal Streptavidin Binding Peptide-Flag (CXCR7-SBP-Flag) epitope (Additional file 1: Figure S1.B, lane 2) [42], and the second derivative was the SBP cell line, which stably expresses the C-terminal SBP-Flag epitope (Additional file 1: Figure S1.B, lane 1). As predicted, pAbCXCR7 cross-reacted with the endogenously expressed CXCR7 protein in both the SBP and C7-SBP cells (*i.e.,* ~40 kDa and ~48 kDa) (Additional file 1: Figure S1.B, left panel). More importantly, the banding pattern for the CXCR7-SBP-Flag fusion protein in the C7-SBP cells was nearly identical between the pAbCXCR7 and anti-SBP antibodies (Additional file 1: Figure S1.B, left panel-pAbCXCR7; right panel-SBP). These findings showed that pAbCXCR7 recognized heterologously expressed, epitope-tagged CXCR7 in LNCaP cells, and that pAbCXCR7 specifically recognized the endogenous CXCR7 protein in these cells.

We also examined expression of the CXCR7 protein in human epithelial cancers, since the transcripts are expressed in human transformed cell lines [43]. Western blot analysis showed the 40- and 48-kDa CXCR7 isoforms were expressed in the panel of neoplastic human epithelial cell lines (Additional file 1: Figure S1.C). This included the human prostate-cancer cell lines LNCaP, 22Rv.1, DU145, and PC3, the human breast cancer line MCF7, the human cervical cancer line HeLa, and the human embryonic kidney line HEK293 (Additional file 1: Figure S1.C). These results confirmed that CXCR7 protein is expressed in both normal adult human tissues and cancerous human epithelial cell lines, as reported previously [41,44,45].

### Intracellular localization of CXCR7 in prostate-cancer cells

**Figure 2:**
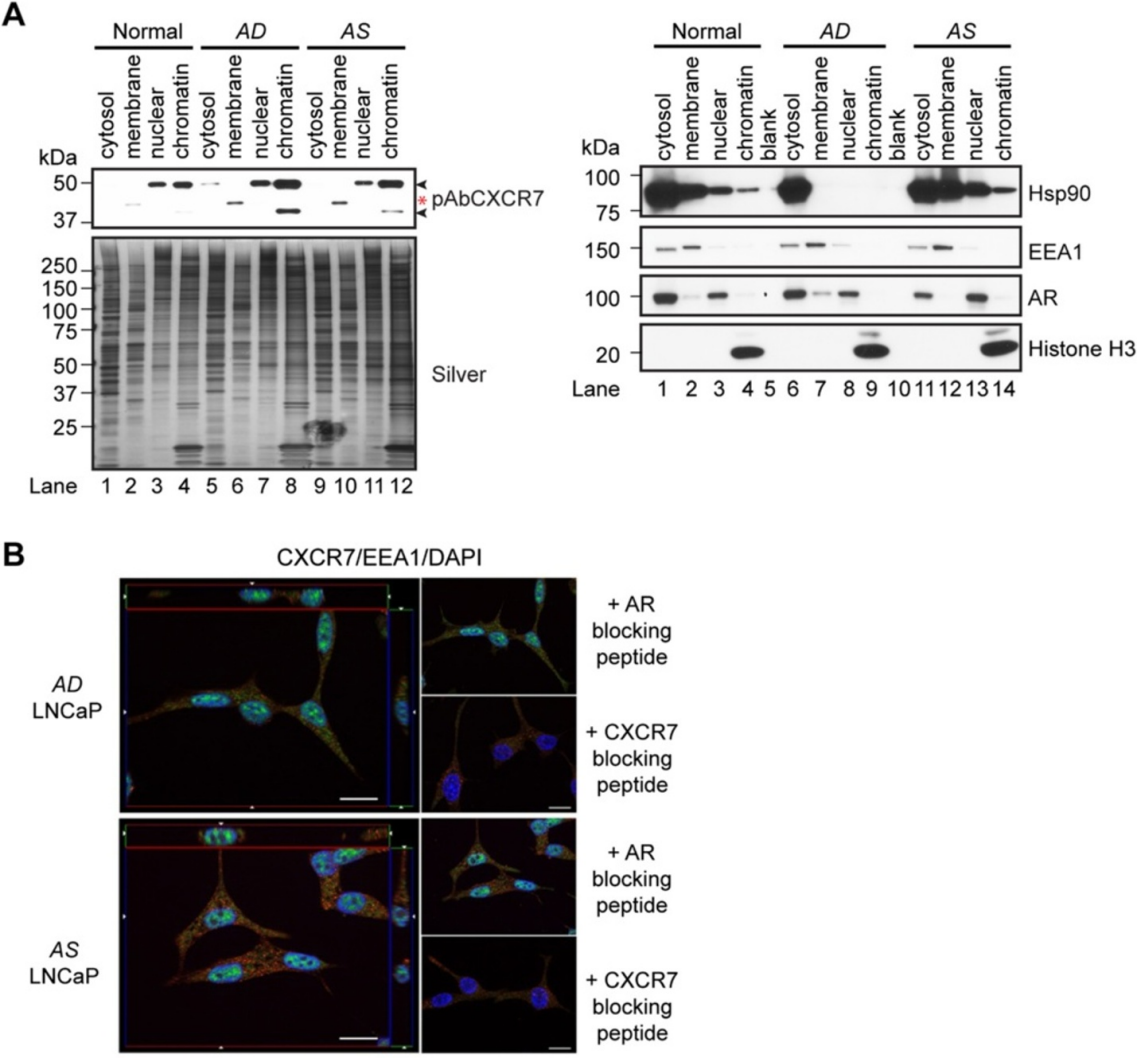
**CXCR7 subcellular localization in prostate-cancer cells. (A)** Western blot of proteins from cytosolic, membrane, nuclear, and chromatin fractions isolated from normal, *AD*-, and *AS*-LNCaP cells with antibodies to pAbCXCR7 (top left panel), Hsp90, EEA1, AR, or histone H3 (right panels, with blank lanes in lanes 5 and 10). Silver-stained gel demonstrated equal protein loading across samples (bottom left panel). **(B)** Upper panels: *AD*-LNCaP cells. Left panel: Image of a single optical section (section 17 out of 32), with “Cut View” analysis of the entire Z-stack shown in margins. Green = CXCR7, Red = EEA1, Blue = DAPI. Right top panel: Reconstruction of entire Z-stack of sample shown in left. Scale bar = 20 μm. Right bottom panel: Similar reconstruction of cells stained in the presence of competing peptide. Scale bar in lower right = 20 μm and is for top and bottom right panels. Lower panels: Androgen stimulated LNCaP cells. Left panel: Image of a single optical section (section 12 out of 32), with “Cut View” analysis of the entire Z-stack shown in margins. Green = CXCR7, Red = EEA1, Blue = DAPI. Scale bar = 20 μm. Right top panel: Reconstruction of entire Z-stack of sample shown in left. Right bottom panel: Similar reconstruction of cells stained in the presence of competing peptide. Scale bar in lower right = 20 μm and is for top and bottom right panels.

CXCR7 has been localized to the cell surface of neoplastic prostate epithelial cells [41,46], but the intracellular localization of endogenously expressed CXCR7 remains poorly defined. We used indirect immunofluorescence (IF) to characterize the intracellular localization of CXCR7 protein in both androgen-sensitive (*i.e.*, LNCaP, 22Rv1) and androgen-refractory (*i.e.*, PC-3, DU145) human prostate tumor cell lines. Chronic exposure of LNCaP cells to synthetic androgen R1881 has been shown to reduce CXCR7 levels in microsomes [40], and thus we wanted to characterize the intracellular localization of CXCR7 in the absence or presence of androgen. Western blot analysis and IF analysis was performed on LNCaP cells grown in normal, androgen-depleted (*AD*), or androgen-stimulated (*AS*) growth medium (Figure 2). First, we assessed the influence of androgens on CXCR7 levels and/or subcellular localization in LNCaP cells. A detergent-based kit was used to generate cytosolic, membrane, nuclear, and chromatin protein extracts from normal, *AD*-, and *AS*-LNCaP cells. The intensity of the ~48 kDa CXCR7 isoform was increased in the cytosolic, nuclear, and chromatin fractions from the *AD*-cells relative to *AS* and normal cells (Figure 2A, compare lanes 1,3-4, 5, 7–8, 9, 11–12). Although the ~40 kDa CXCR7 isoform was undetectable in the cytosolic, membrane, and nuclear fractions, in the case of the chromatin fraction a similar increase was observed for the *AD*–LNCaP cells relative to the *AS*-LNCaP cells and normal cells (Figure 2A, compare lanes 4, 8, and 12). Interestingly, a novel immunoreactive ~44 kDa CXCR7 band was also observed in the membrane fractions of both the *AD*- and *AS*-LNCaP cells (Figure 2A, lane 2, 6, and 10). This led us to verify the integrity of cytosolic, membrane, nuclear, and chromatin-bound protein fractions by subjecting all protein fractions to western blot analysis with the following compartment-specific markers: heat shock protein 90 beta (Hsp90, cytosol), early endosome antigen 1 (EEA1, membrane), androgen receptor (AR, nucleus), and histone H3 (H3, chromatin) (Figure 2A, right panel). EEA1 was primarily present in the cytosolic and membrane fractions, and histone H3 was restricted to the chromatin-bound fractions (Figure 2A, right panel). In mammalian systems, androgens promote the translocation of AR and Hsp90 from the cytoplasm to the nucleus. As predicted, in the cases of both the cytosolic and membrane fractions, AR and Hsp90 levels were increased in the *AD-*cells relative to *AS-*LNCaP cells (Figure 2A, compare lanes 6–7, *AD*-LNCaP cells, to lanes 11–12, *AS* cells). In contrast, in the nuclear fractions, AR levels were increased in the *AS* cells relative to *AD*-cells (Figure 2A, compare lanes 13, *AS-*LNCaP cells, to 7, *AD-*LNCaP cells). In the context of androgen deprivation, Hsp90 levels were restricted to the cytosolic fraction, whereas under androgen stimulation, Hsp90 levels increased in the membrane, nuclear, and chromatin-bound fractions (Figure 2A, compare lanes 6–9 to lanes 11–14). Overall, these results verified the compartment-specific protein localization observed in the fractionated protein extracts, and thus, their utility for verifying the subcellular compartmentalization of CXCR7 in LNCaP prostate-cancer cells.

Next, optical sectioning was used in conjunction with structured illumination microscopy to more precisely delineate the intracellular expression of CXCR7 (Figure 2B). We stained both *AD-* and *AS-* LNCaP cells for CXCR7 and EEA1, and applied DAPI. In both cell types, CXCR7 (green) was found throughout the cytoplasm in puncta that are distinct from the early endosomes (red). Very little plasma membrane-associated staining was observed (lack of staining at the cell periphery) in individual optical sections of the cells, cross sections of the cells, or reconstructions of whole cells (Figure 2B). Notably, both *AD-* and *AS-*LNCaP cells exhibited robust nuclear CXCR7 staining, but the cytoplasmic puncta were more intensely stained in *AD*- vs. *AS-*LNCaP cells (Figure 2B). This was consistent with previously published quantitative protein profiling of microsomes in *AD*- and *AS-*LNCaP cells [40]. The CXCR7 staining in the cytoplasmic and nuclear compartments was specific, as pre-absorption of pAbCXCR7 with the C-terminal CXCR7 peptide eliminated intracellular CXCR7 staining (Figure 2B). These results showed that CXCR7 is present in the cytoplasm and nucleus in both *AD*- and *AS-*LNCaP prostate-cancer cells.

Further IF analyses in 22Rv1, PC-3, and DU145 prostate-cancer cells showed that CXCR7 was localized to the membrane-cytoplasmic and nuclear compartments (Additional file 1: Figure S1.D, I-I, II-I, and III-I), whereas in DU145 prostate-cancer cells it was restricted to the nuclear compartment (Additional file 1: Figure S1.D, II-I). Importantly, staining was specific for CXCR7, as CXCR7 immunoreactivity was abolished in LNCaP, 22Rv1, DU145, and PC3 cells when pAbCXCR7 was pre-absorbed to the C-terminal CXCR7 peptide (Figure 2B, *AD*, *AS* lower right panels, and Additional file 1: Figure S1.D, I-III, II-III, and III-III). These results demonstrated that intracellular CXCR7 is present in both the cytoplasm and nucleus of human prostate-cancer cells.

### CXCL11 and CXCL12 modulate the expression of CXCR7, CXCR4, and AR in LNCaP cells

**Figure 3:**
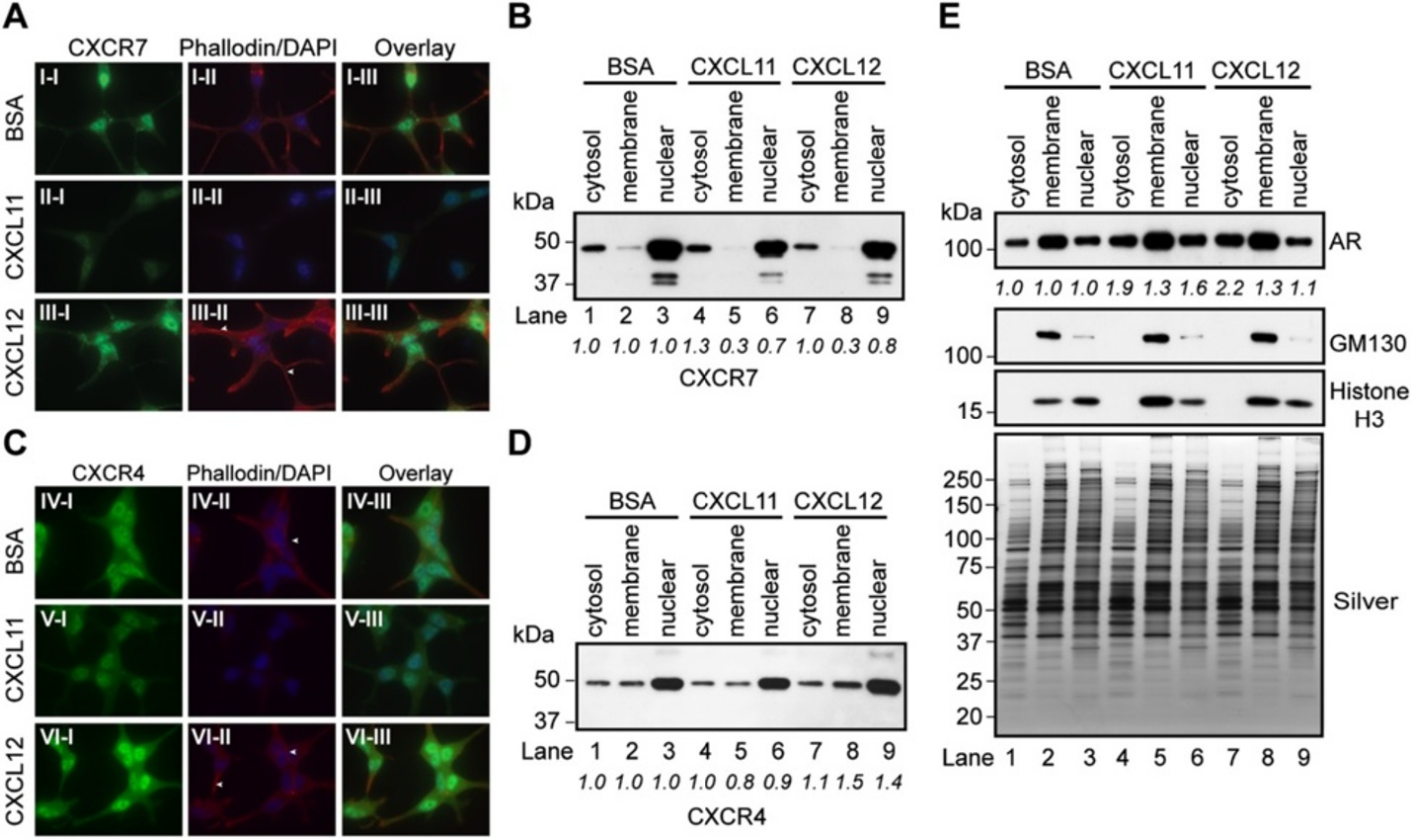
**CXCR7 expression and localization are modulated by CXCL11 and CXCL12. (A)** Immunofluorescence staining of CXCR7 in *AD*-LNCaP cells treated with vehicle (0.1% BSA), CXCL11 (100 nM), or CXCL12 (100 nM) for 30 min. Nuclei and F-actin are labeled with DAPI and Texas-red phalloidin, respectively. **(B)** Western blot of cytosolic, membrane, and nuclear protein fractions isolated from LNCaP cells, cultured as described in **(A)** with pAbCXCR7 antibody. **(C)** Immunofluorescence staining of CXCR4 in *AD*-LNCaP cells as described in **(A)**. **(D-E)** Western blot of cytosolic, membrane, and nuclear protein fractions isolated from LNCaP cells, cultured as described in (A), with antibodies to **(D)** CXCR4, and **(E)** AR, GM130, and histone H3. Silver staining demonstrates equivalent loading across samples. The densitometry values were normalized to BSA-treated samples for each subcellular compartment and labeled below the blots.

In prostate-cancer cells, androgens are known to stimulate expression of the CXCR4 mRNA and to repress that of the CXCR7 mRNA [8,25,47]. Moreover, androgen-triggered motility of prostate-cancer cells depends on signaling by the CXCL12/CXCR7/CXCR4 axis [8,25]. We thus tested whether acute exposure to either CXCL11 or CXCL12 affects the levels of CXCR7 or its intracellular localization in *AD*-LNCaP cells. The cells were incubated with bovine serum albumin (BSA) (control), CXCL11 (100 nM), or CXCL12 (100 nM) for 30 mins and processed for IF analysis. CXCR7 expression (green), with respect to the cytoplasm (F-actin as labeled with Texas Red-X Phalloidin) and nucleus (DAPI), was then evaluated (Figure 3A). CXCL11 treatment resulted in a low CXCR7 immunoreactivity in the cytoplasm and nucleus relative to that in control (BSA-treated) cells (Figure 3A, compare II-I to I-I). In contrast, acute treatment with CXCL12 led to an increase in the CXCR7 signal in both the cytoplasmic and nuclear compartments (Figure 3A, compare III-I to I-I and II-I). Notably, CXCR7 staining was concentrated in the cytoplasmic puncta of CXCL12-treated cells. This finding suggests that CXCL12-mediated binding to the CXCR7 and/or CXCR4 receptors induces the formation of cytoplasmic puncta, possibly by mobilizing plasma membrane-bound or intracellular CXCR7 (Figure 3A, III-I).

We extended the IF analysis to CXCR4 to determine whether the effects of CXCL11 and CXCL12 were restricted to CXCR7. Since CXCR4 and CXCR7 form heterodimers that promote cell migration in response to CXCL12 stimulation [17,18,48,49], we assessed CXCR4 levels and compartmentalization after acute treatment with CXCL12. Intracellular CXCR4 was detected using the anti-CXCR4 antibody ab2074, which recognizes extracellular N-terminal residues (1–14) of the human protein. In control cells, intracellular CXCR4 staining was diffuse in both the cytoplasm and the nucleus (Figure 3C, IV-I). Notably, in CXCL11-treated cells, CXCR4 staining was lower in both the cytoplasm and nucleus (Figure 3C, compare V-I to IV-I), concordant with the reduction in intracellular CXCR7 observed in CXCL11-treated cells (Figure 3A, II-I). In CXCL12-treated cells, by contrast, CXCR4 staining was increased in the cytoplasmic and nuclear compartments (Figure 3C, compare VI-I to IV-I), concordant with an increase in CXCR7 staining in CXCL12-treated cells (Figure 3A, III-I). Analysis of the expression of F-actin revealed that its levels were increased in CXCL12-treated cells (Figure 3A, compare III-II to I-II; 3C, VI-II to IV-II, respectively) and decreased in CXCL11-treated cells (Figure 3A, compare II-II to I-II; 3C, compare V-II to IV-II). Thus, F-actin polymerization was differentially regulated by CXCL12 and CXCL11 in *AD-*LNCaP cells. The stimulation of F-actin polymerization in the context of CXCL12 in these cells was reminiscent of that observed during CXCL12-mediated cell motility of HCT116 colon-carcinoma cells [50]. Together, these data showed that acute stimulation by chemokines CXCL11 and CXCL12 changed the intracellular immunoreactivity of CXCR4 and CXCR7 in *AD*-LNCaP prostate-cancer cells.

Next, we sought to establish whether the observed effects of chemokines on the intracellular localization of CXCR4 and CXCR7 was a consequence of epitope masking or *bona fide* changes in either their abundance at the protein level and/or their compartmentalization. To measure CXCR4 and CXCR7 levels across the intracellular compartments, we performed western blot analysis on cytosolic, membrane, and nuclear protein extracts from *AD*-LNCaP cells exposed to CXCL11 and CXCL12 (Figure 3B and D). The 40- and 48-kDa CXCR7 isoforms were detected across the cytosolic, membrane, and nuclear fractions (Figure 3B). Both CXCL11 and CXCL12 reduced levels of the 48-kDa isoform in the nuclear and membrane fractions and the levels of the 40-kDa isoform in the nuclear fraction (Figure 3B, compare lanes 2, 3, 5, 6, 8 and 9). Similar to CXCR7, CXCR4 was detected across the cytosolic, membrane, and nuclear fractions of all experiment groups (Figure 3D). However, CXCL11 treatments had minimal effects on levels of the ~50 kDa CXCR4 receptor relative to those in the BSA-treated cells (Figure 3D, compare lanes 1–6), whereas CXCL12 increased CXCR4 levels in the membrane and nuclear fractions (Figure 3D, compare lanes 2–3, and 8–9). Overall, these results show that under conditions of androgen depletion, acute stimulation with CXCL11 or CXCL12 leads to a reduction in the intracellular levels of CXCR7, while stimulation with CXCL12 increases CXCR4 levels.

Chronic CXCL12 exposure has recently been shown to promote androgen-independent but AR-dependent proliferation by LNCaP cells [51]. Therefore, we assessed whether CXCL11 or CXCL12 had any effect on the levels or subcellular localization of AR. Western blot analysis showed that AR levels increased in the cytosolic, membrane, and nuclear fractions of CXCL11-treated cells relative to control cells (Figure 3E, first panel: compare lanes 1–6). In addition, CXCL12 treatment increased AR levels in the cytosolic and membrane fractions but not the nuclear fractions (Figure 3E, first panel: compare lanes 1–3, 7–9). To ensure that acute exposure to CXCL11 and CXCL12 had no effect on proteins that are unrelated to chemokine-mediated signaling, we verified the integrity of the cytosolic, membrane, and nuclear fractions. The compartment-specific marker proteins selected were the Golgi matrix protein of 130 kD (GM130) and the chromatin-associated histone H3 (Figure 3E, second panel: GM130; third panel: histone H3). Histone H3 was predominantly in the nuclear fraction of BSA-treated cells, however, exposure to both CXCL11 and CXCL12 increased histone H3 levels in the membrane fraction and marginally decreased its levels in the nuclear fraction (Figure 3E, third panel: compare lanes 2–3, 5–6, and 8–9). Given these were crude protein extracts, we suspect the residual histone staining was due to potential leakage or cross-contamination of the Histone H3 proteins into the crude membrane fraction. The purity of the nuclear fraction was confirmed as GM130 was predominantly localized to the membrane fraction, and its levels were slightly increased in the membrane fractions of both CXCL11- and CXCL12-treated cells relative to control cells (Figure 3E, second panel: compare lanes 2, 5, and 8). Overall, these findings demonstrate that acute stimulation by chemokines CXCL11 and CXCL12 influences intracellular protein metabolism and/or protein trafficking in *AD*-LNCaP prostate-cancer cells.

### Chemokines 11 and 12 modulate androgen-regulated gene expression in LNCaP cells

**Figure 4:**
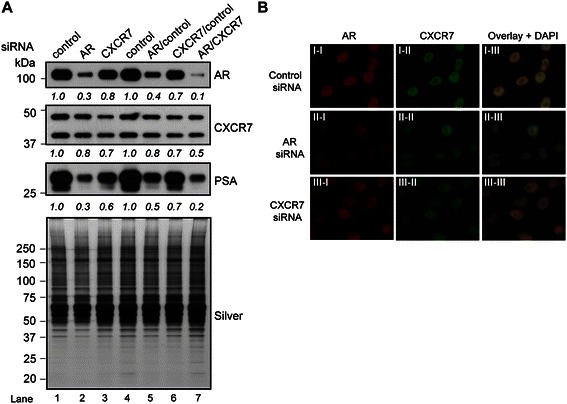
**CXCR7 functionally interacts and colocalizes with AR. (A)** Western blot of LNCaP cells transfected with the indicated siRNA combinations: control (50 nM), AR (50 nM), CXCR7 (50 nM), AR/control (25 nM/25 nM), CXCR7/control (25 nM/25 nM), or AR/CXCR7 (25 nM/25 nM) for 72 hrs. Western blot was performed using AR, CXCR7, and PSA antibodies. Silver staining demonstrates equivalent loading across the samples. The densitometry values were normalized to control siRNA transfected cells and labeled below the blots. **(B)** Immunofluorescence analysis of CXCR7 and AR in LNCaP cells under AR or CXCR7 knockdown conditions. Cells were transfected with control (I-I to I-III), AR (II-I to II-III), or CXCR7 (III-I to III-III) siRNA, stained with antibodies against AR and CXCR7, and treated with DAPI.

The CXCL12/CXCR4 axis engages the AR signaling pathway by promoting ligand-independent AR activity in LNCaP cells [51]. We thus reasoned that the CXCL11/CXCR7 axis may also engage the AR signaling pathway in human prostate-cancer cells. Therefore, we examined potential functional interactions between CXCR7 and AR that could explain how AR levels and/or localization were modulated by CXCL11 and CXCL12. First, we wanted to determine if CXCR7 expression is required for the normal transcriptional activity of AR in LNCaP cells. Western blot analysis was carried out on extracts from cells transfected with control, AR, and CXCR7 siRNAs, and the expression of PSA, a model androgen-regulated gene that serves as a surrogate marker of AR transcriptional activity in prostate-cancer cells, was monitored [51,52]. The levels of CXCR7 and PSA were reduced in CXCR7 knockdown cells (50 nM siRNA for 96 hrs) relative to control cells (Figure 4A, second panel: compare lane 1 and 3, third panel: compare lane 1 and 3), concordant with the expected reduction in PSA levels that was observed in AR knockdown cells (Figure 4A, third panel: compare lane 1 and 2). Thus, CXCR7 expression was required for normal AR activity in LNCaP cells. Interestingly, CXCR7 levels were reduced in AR knockdown cells (Figure 4A, second panel: compare lane 1 and 2), and AR levels were reduced in CXCR7 knockdown cells (Figure 4A, first panel: compare lanes 1 and 3).

Having shown that CXCR7 is required for the normal expression of AR and PSA in LNCaP cells, we sought to explore a genetic interaction between CXCR7 and AR in the context of AR signaling [51]. CXCR7 and AR were co-targeted for siRNA-mediated knockdown, and AR and PSA expression was evaluated by western blot analysis (Figure 4A, first and third panels: compare lanes 4–7). As expected, AR and CXCR7 levels were reduced in cells co-transfected with AR and control siRNAs (i.e., 25 nM/25 nM, 50 nM total) and CXCR7 and control siRNAs relative to cells transfected with control siRNA only (Figure 4A, first and second panels: compare lanes 4–6). Again, we detected a co-dependence in expression between AR and CXCR7; a small reduction in AR levels was observed in CXCR7/control knockdown cells relative to control cells (Figure 4A, first panel: compare lane 4 and 6), and a small reduction in CXCR7 levels was detected in AR/control knockdown relative to control cells (Figure 4A, second panel: compare lane 4 and 5). More importantly, comparison of AR/CXCR7 knockdown to AR/control, CXCR7/control, and control knockdown cells revealed an additive reduction in AR and PSA levels (Figure 4A, first, and third panels: compare lanes 4–7).

Next, we examined the colocalization of CXCR7 and AR in LNCaP cells in normal growth medium, since CXCR7 and AR were found in the same cellular fractions (Figure 3B, 3E). IF staining of control siRNA-transfected cells showed strong nuclear staining for both AR (red) and CXCR7 (green), and this staining was not present in cells transfected with AR- and CXCR7-targeted siRNAs (Figure 4B, compare I-I to II-I and III-I, and I-II to II-II and II-III). Overlay of the AR and CXCR7 channels for control cells revealed a strong yellow staining pattern (Figure 4B, I-III). In addition, overlay of the AR and CXCR7 channels in cells transfected with either an AR or CXCR7 siRNA showed a reduction in intensity of the yellow staining pattern, supporting the notion that AR and CXCR7 are co-localized in the nucleus. (Figure 4B, compare I-III to II-III and III-III). Moreover, these results were congruent with the western blot results (Figure 4A), and suggest that CXCR7 and AR expression are co-regulated in LNCaP cells.

### Colocalization and physical interaction of AR and CXCR7-SBP in LNCaP cells

**Figure 5:**
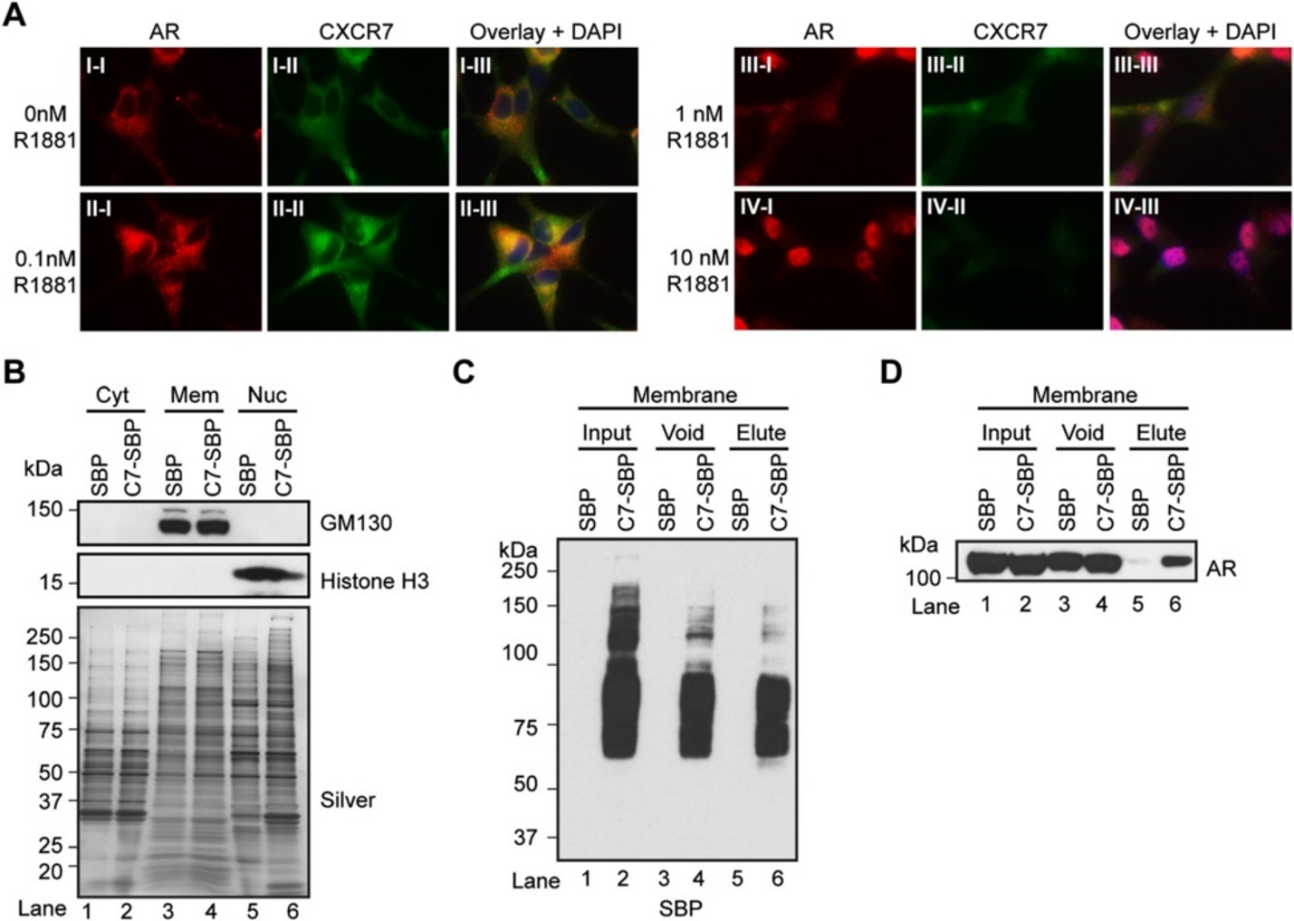
**CXCR7 colocalizes and physically interacts with AR in LNCaP prostate-tumor cells. (A)** Immunofluorescence staining of CXCR7 and AR in semi-permeabilized *AD*-LNCaP cells treated with vehicle (0.1% BSA), 0.1, 1, or 10 nM R1881 for 96 hrs. The nuclei are labeled with DAPI. **(B)** Western blot analysis of crude cytosolic, membrane, and nuclear proteins isolated from LNCaP cells stably expressing streptavidin binding peptide tag (SBP) or CXCR7 with a SBP-tag on the C-terminus (C7-SBP) using antibodies to GM130 and Histone H3 (top panel). Silver-stained gel demonstrated equal protein loading across samples (bottom panel). **(C)** Western blot analysis of streptavidin affinity purified samples from detergent-solubilized microsomal protein fraction of SBP or C7-SBP cells using the SBP, and **(D)** AR antibody. Equal proportions (10 μl) of input, flow-through (void), and 5% (10 ul) of the biotin elution were loaded.

These results prompted us to further explore the molecular relationship between AR and CXCR7 because an additive interaction on AR, PSA, and CXCR7 expression was observed in AR/CXCR7 double knockdown cells [52]. Therefore, we explored if AR and CXCR7 were further co-localized beyond the nuclear compartment to include the cytosol and membrane compartments in LNCaP cells. Thus, AR and CXCR7 were co-stained in the cytosolic and membrane compartments of semi-permeabilized (*i.e.*, 4% formalin and 4% methanol) LNCaP cells in response to different doses of androgen for 24 hrs. AR staining was restricted to the cytosolic and membrane compartments in *AD*-LNCaP cells treated with vehicle (ethanol), while staining in the cytosolic and Golgi-like compartments increased in a dose-dependent manner with androgen (Figure 5A, I-I, II-I, III-I). However, AR staining was nearly undetectable in the cytosolic and membrane compartments and was restricted to the nuclear compartment at the highest dose of androgen (Figure 5A, IV-I). Similarly to AR, androgen increased CXCR7 staining in the cytosolic and membrane compartments in a dose-dependent manner until staining was completely undetectable at the highest dose of androgen (Figure 5A, I-II, II-II, III-II, and IV-II). More importantly, overlay of the AR and CXCR7 channels showed they were co-localized in the cytosolic and membrane compartments in vehicle- and 0.1nM R1881-treated *AD*-LNCaP cells (Figure 5A, I-III, II-III). These results demonstrate that androgens had a dose-dependent effect on the co-localization of AR and CXCR7 in LNCaP cells to suggest a potential physical interaction between AR and CXCR7 in LNCaP cells.

Next, we wanted to determine if CXCR7 and AR physically interacted in LNCaP cells. We decided to test if endogenous AR physically interacted with CXCR7-SBP in C7-SBP cells because the SBP epitope provided a high-affinity tag for the selective isolation of the CXCR7-SBP protein using streptavidin-affinity chromatography [42]. Since a strong co-localization signal between AR and CXCR7 was detected in the membrane fraction of LNCaP cells (Figure 5A), we explored a physical interaction between CXCR7-SBP and AR in the detergent-solubilized microsomal protein fraction of CXCR7-SBP cells relative to SBP cells using streptavidin-affinity chromatography (Figure 5C-5D). Western blot analysis of crude cytosolic, membrane, and nuclear protein fractions verified the compartmentalization of known subcellular protein markers (Figure 5B, upper panel- membrane marker GM130, middle panel- nuclear marker Histone H3, lower panel silver-stained gel). As shown in Figure 5C, SBP western blot analysis demonstrated the affinity-purification and biotin elution of CXCR7-SBP from C7-SBP cells relative to SBP cells (Figure 5C, compare lanes 5 and 6). More importantly, western bot analysis showed AR was selectively enriched in the biotin-eluted microsomal protein extracts derived from C7-SBP cells relative to SBP cells (Figure 5D, compare lanes 5 and 6). These biochemical results demonstrated the co-elution of CXCR7-SBP and AR proteins to support a physical interaction between CXCR7-SBP and AR in the membrane fraction of C7-SBP cells.

**Figure 6:**
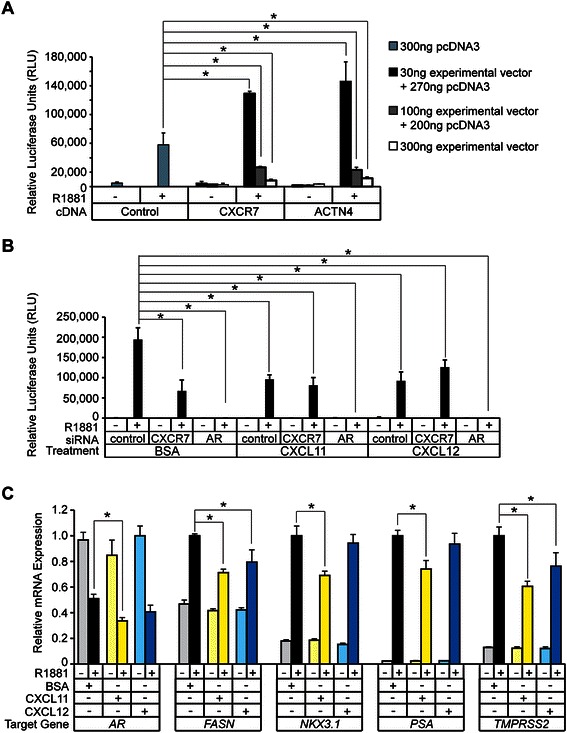
**CXCR7 modulates AR transcriptional activity. (A)** Luciferase assay testing the effects of CXCR7 overexpression on the AR-target promoter *probasin* in LNCaP cells. LNCaP cells were co-transfected with the pGL4.10-Luc2-*probasin* and pRLSV40 *Renilla* vectors along with increasing amounts (30 ng experimental + 270 ng pcDNA3, 100 ng experimental + 200 ng pcDNA3, 300 ng experimental + 0 ng pcDNA3) of CXCR7 or ACTN4 cDNA mammalian expression vectors. The maximal amount (300 ng) of the pcDNA3 mammalian expression vector served as the positive control. Cells were subsequently treated with androgen (1 nM R1881) or vehicle (ethanol) and tested for dual luciferase activity. Student’s *t*-test was used to calculate significant differences (*p ≤ 0.05, n = 3) between control and experimental cells within the androgen-treatment group. **(B)** Luciferase assay testing the effects of CXCR7 siRNA knockdown and treatment with CXCR7 ligand on the AR-target promoter *probasin*. LNCaP cells were co-transfected with the pGL4.10-Luc2-*probasin* and pRLSV40-*renilla* vectors, along with control or experimental siRNAs (50 nM). Next, cells were pre-treated with indicated ligands (BSA, CXCL11, or CXCL12) for 30 min. Cells were then subsequently treated with androgen (1 nM R1881) or vehicle (ethanol) for 18 hrs and tested for dual luciferase activity. Student’s *t*-test was used to calculate significant differences (*p ≤ 0.05, n = 3) between control cells and experimental cells within the androgen-treatment group. **(C)** RNA isolated from LNCaP cells treated with vehicle (0.1% BSA), CXCL11 (10 nM), or CXCL12 (10 nM) for 30 min and subsequently treated with vehicle or androgen (1 nM R1881) for 18 hrs were subjected to qPCR analysis for *AR*, *FASN*, *NKX3.1*, *PSA*, and *TMPRSS2* gene expressions. Student’s *t*-test was used to calculate significant differences (*p ≤ 0.05, n = 3) between control and chemokine ligand-treated cells.

Since a physical interaction between CXCR7-SBP and AR was detected in the membrane fraction of C7-SBP cells (Figure 5C and D), and CXCR7 expression was required for optimal AR and PSA expression (Figure 4A), we assessed the effects of CXCR7 overexpression on AR-mediated transcription in LNCaP cells. Endogenous AR activity was measured in vehicle- and androgen-treated *AD*-LNCaP cells co-transfected with the androgen-responsive *probasin*-luciferase expression vector and increasing amounts of the CXCR7-SBP expression vector (30 ng, 100 ng, and 300 ng; Figure 6A) [53]. To compare the effects of CXCR7 to that of a known co-regulator of AR-mediated transcription, *AD*-LNCaP cells were co-transfected with an expression vector encoding α-actinin-4, a protein that enhances or represses AR activity when expressed at low and high doses, respectively, in mammalian cells (Figure 6A) [53]. CXCR7 had a dose-dependent effect on AR-mediated transcription in CXCR7-transfected cells (Figure 6A). The lowest dose of CXCR7 expression vector enhanced AR activity relative to that in control samples, while higher doses progressively repressed AR activity (Figure 6A). These results show that CXCR7 can co-regulate AR-mediated transcription in LNCaP cells, thus providing evidence of a functional relationship between CXCR7 and AR in prostate-cancer cells.

This prompted us to further characterize crosstalk between CXCR7 and AR, testing whether pre-treatment of *AD*-LNCaP cells with CXCL11 or CXCL12 could influence androgen-dependent gene transcription through CXCR7 (Figure 6B). Endogenous AR activity was measured 48 hrs post-transfection in *AD*-LNCaP cells cotransfected with the *probasin*-luciferase vector and control, CXCR7, or AR siRNAs. The *AD*-LNCaP cells were treated with BSA (100 nM, control), CXCL11 (100 nM), or CXCL12 (100 nM) for 30 mins, and then challenged with androgen (*i.e.,* 1 nM R1881) for 24 hrs. We found that both CXCL11 and CXCL12 antagonized AR activity in control knockdown cells, demonstrating that both chemokines attenuated androgen-induced AR transcriptional activity (Figure 6B). Moreover, CXCR7 expression was required for optimal AR transcriptional activity; luciferase activity was noticeably reduced in CXCR7 knockdown versus control cells (Figure 6B). Since CXCL11 and CXCL12 both engage CXCR7, these results suggest that CXCR7 expression is required for CXCL11- and CXCL12-mediated antagonism of AR activity in LNCaP cells.

We extended these findings by exploring whether CXCL11 and CXCL12 could modulate the expression of endogenous androgen-regulated genes (*ARGs*) in LNCaP cells [54]. The target *ARGs* included AR, fatty acid synthase (*FASN*), NK3 homeobox 1 (*NKX3.1*), prostate-specific antigen (*PSA*), and transmembrane protease serine 2 (*TMPRSS2*) (Figure 6C). qPCR analysis was performed on 48 hr *AD*-LNCaP cells that were pre-treated with BSA (0.1%, control), CXCL11 (100 nM), or CXCL12 (100 nM) for 30 mins, and subsequently challenged with vehicle (ethanol) or androgen (1 nM R1881) for 24 hrs. CXCL11 antagonized the expression of all *ARGs* in the androgen-treated cells, whereas CXCL12 antagonized the expression of only *FASN* and *TMPRSS2* in this context (Figure 6C). These results demonstrate that the CXCL11 and CXCL12 signaling pathways intersect with the AR-regulated gene expression program in LNCaP cells.

### Androgens regulate CXCR4 and CXCR7 expression and potential glycosylation linked to cell motility in LNCaP cells

**Figure 7:**
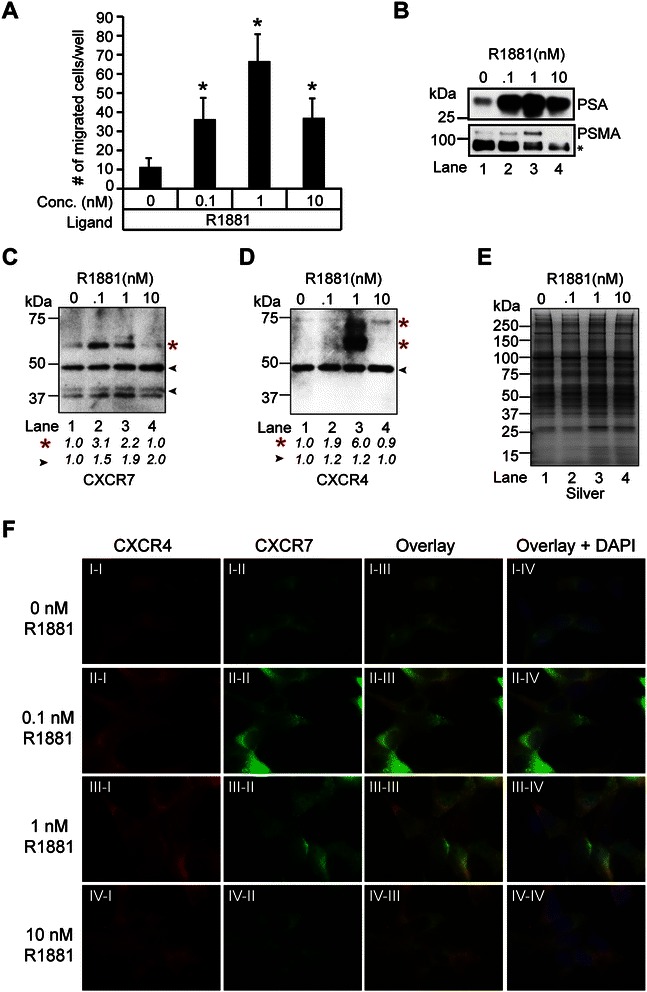
**CXCR7 modulates androgen-mediated cell motility through CXCR4. (A)** Transwell assay assessing the effects of androgens on LNCaP migration. *ANOVA* was used to determine significant differences between vehicle (ethanol) and androgen (R1881) treated cells (*p ≤ 0.05, n = 3). (B-D) Western blot analysis of LNCaP membrane glycoproteins enriched from cells grown in androgen-depleted medium for 72 hrs and treated with 0, 0.1, 1, or 10 nM R1881 for 24 hrs with antibodies to **(B)** PSA, PSMA, **(C)** CXCR7, and **(D)** CXCR4. Red asterisks = putative glycosylated CXCR4 and CXCR7 isoforms. The densitometry values were normalized to vehicle-treated lysates and labeled below the blots. **(E)** Silver staining demonstrates equivalent loading across the samples. **(F)** Immunofluorescence staining of CXCR4 and CXCR7 in semi-permeabilized *AD*-LNCaP cells treated with 0, 0.1, 1, or 10 nM R1881. The nuclei are labeled with DAPI.

CXCR7 regulates CXCL12/CXCR4-mediated cell motility by scavenging CXCL12, both during normal processes (*e.g.,* development) and in the diseased state (*e.g.,* cancer). CXCR7 can act in a cell-autonomous manner in cells that co-express CXCR4, or *in trans* when CXCR4 expression is restricted to adjacent cells [37]. We wanted to determine if CXCR7 is a critical determinant of androgen-mediated cell motility in LNCaP cells, since androgens stimulate cell motility through KLF5-mediated upregulation of CXCR4 transcription [25]. First, we wanted to determine if there is an androgen concentration that stimulates maximal cell motility in LNCaP cells. A bare filter cell migration assay was performed on LNCaP cells treated with vehicle (ethanol) or androgen at various concentrations (*i.e.,* 0.1 nM, 1 nM, and 10 nM R1881). Increasing concentrations of androgen produced a biphasic cell motility response in LNCaP cells, and the maximum response was observed at 1 nM R1881 (Figure 7A). This increase in cell motility was not due to an increase in cell proliferation (Additional file 2: Figure S2.B). These findings demonstrate that the androgen-mediated cell motility of LNCaP prostate-cancer cells is dose-dependent.

Since androgens promote cell motility through the upregulation of CXCR4 in LNCaP cells [8,25], we wanted to examine how different doses of androgens influence the expression of CXCR4 and CXCR7 in the context of androgen-mediated cell motility. To isolate mature receptors capable of binding soluble CXCL12 ligand at the plasma membrane, we implemented a purification strategy that enabled us to isolate glycosylated isoforms of CXCR4 and CXCR7. Detergent solubilized microsomal protein extracts from 72 hr *AD*-LNCaP cells challenged with vehicle (ethanol), 0.1 nM, 1 nM, or 10 nM R1881 for 24 hrs were subjected to lectin affinity chromatography (*i.e.*, wheat-germ agglutinin (WGA) and concanavalin A (ConA)) and selectively eluted with N-acetylglucosamine and mannose sugars to isolate N-linked and O-linked membrane and membrane-associated glycoproteins. To validate the enrichment of known androgen-regulated membrane and membrane-associated glycoproteins, western blot analysis of LNCaP lysates was performed for expression of the model androgen-regulated glycoproteins PSMA and PSA (Figure 7B). Consistent with the known repression of PSMA expression by androgens [54], a dose-dependent decrease in glycosylated PSMA was observed with higher doses of androgen (Figure 7B). PSA expression, in contrast, is stimulated by androgens [55,56], and as expected, a dose-dependent increase in glycosylated PSA was observed up to the 1 nM dose of androgen, although a reduction was detected in cells treated with 10 nM androgen (Figure 7B). We extended the western blot analysis to CXCR7 and CXCR4. Even though the 40- and 48-kDa CXCR7 isoforms were present at each dose of androgen (Figure 7C), a larger molecular weight (putatively glycosylated) isoform of CXCR7 at ~60 kDa was detected predominantly in the 0.1 nM R1881-treated sample (Figure 7C). Similarly to CXCR7, the predicted ~50-kDa CXCR4 isoform was present at each dose of androgen (Figure 7D*).* However, several higher molecular-weight immunoreactive CXCR4 isoforms were also detected in the 1 nM R1881-treated sample, suggesting that these are glycosylated isoforms of CXCR4 (*i.e.*, ~60-, 75-kDa) (Figure 7D, lane 3). These findings validate the dose-dependent effects that androgens have on the glycosylation of androgen-regulated proteins in LNCaP prostate-cancer cells. Moreover, these results also suggest that CXCR4 and CXCR7 are potential androgen-regulated glycoproteins in LNCaP prostate-cancer cells.

Next, we wanted to examine, in greater detail, the dose-dependent effects of androgens on the expression of cell surface- or membrane-localized CXCR4 and CXCR7 proteins in LNCaP cells. IF analysis of CXCR4 and CXCR7 was performed on 72 hr *AD*-LNCaP cells subjected to control treatment (ethanol) or exposed to various doses of androgens (*i.e.*, 0.1, 1, 10 nM R1881) for 24 hrs (Figure 7F). To minimize nuclear staining of CXCR4 and CXCR7, cells were semi-permeabilized to better evaluate CXCR4 and CXCR7 staining at the plasma membrane, membrane-associated structures, and cytosolic compartments in both *AD*- and *AS-*LNCaP cells. CXCR4 protein was faintly detectable along the plasma membrane and punctate intracellular structures in vehicle and androgen-treated *AD*-LNCaP cells (Figure 7F, I-I, II-I, III-I, IV-I), while CXCR7 exhibited strong perinuclear staining (Figure 7F, I-II, II-II, III-II, IV-II). CXCR4 staining increased at the plasma membrane and perinuclear compartment at concentrations of up to the 1.0 nM androgen, but was undetectable at the 10 nM concentration (Figure 7F, IV-I). The intracellular and perinuclear staining of CXCR7 were visibly increased at 0.1 nM androgen but noticeably reduced at the higher doses (1 and 10 nM R1881; Figure 7F, III-II and IV-II). More importantly, an overlapping staining pattern for CXCR4 and CXCR7 was detected in the perinuclear compartment, and this co-localization was maximal at 1 nM androgen (Figure 7F, III-III). Interestingly, this dose of androgen triggers maximal androgen-mediated cell motility response in LNCaP cells to support current models that cellular motility is regulated through functional CXCR4/CXCR7 heterodimers (Figure 7A) [37,49]. Thus, androgens have dose-dependent effects on the intracellular localization and co-localization of CXCR4 and CXCR7 proteins in LNCaP prostate-cancer cells.

### CXCR7 regulates the androgen-mediated motility in LNCaP cells

**Figure 8:**
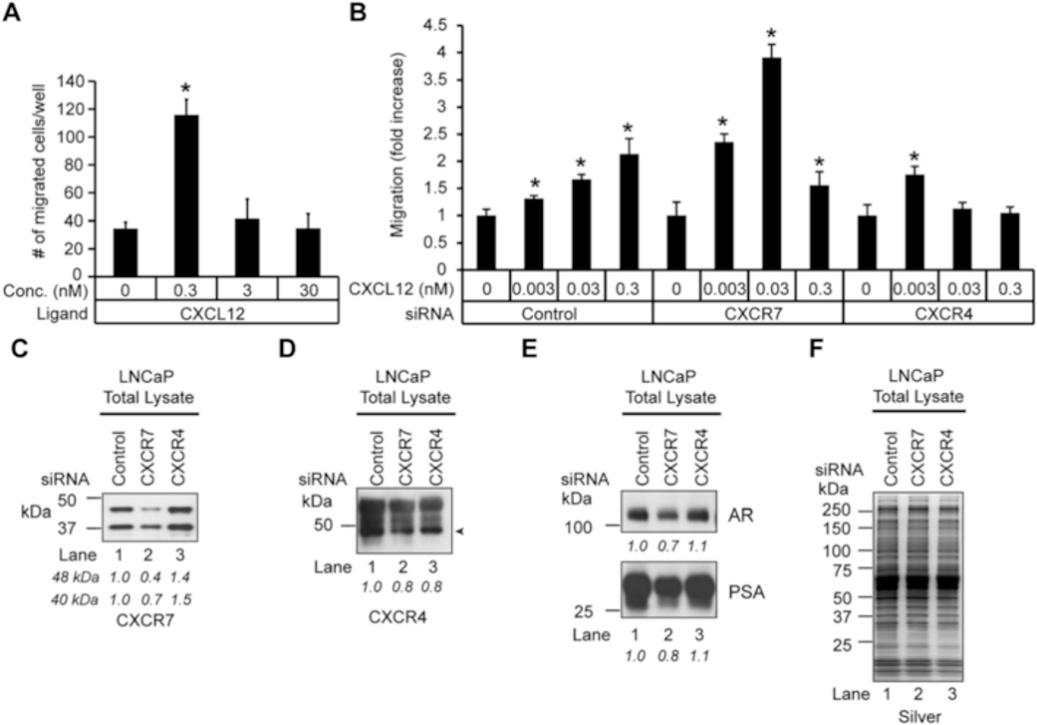
**CXCR7 knockdown leads to a reduction in CXCR4 protein levels in LNCaP cells. (A)** Transwell assay assessing the effects of CXCL12 on LNCaP migration. *ANOVA* was used to determine significant differences between vehicle (0.1% BSA) and CXCL12-treated cells (*p ≤ 0.05, n = 3). **(B)** Transwell assay assessing the effects of CXCR4 and CXCR7 knockdown on CXCL12-induced LNCaP cell migration. LNCaP cells transfected with 100 nM scrambled control, CXCR4, or CXCR7 siRNAs were seeded to the top chamber of the insert. The bottom chamber contained medium with 1% CS serum with 1 nM R1881 and CXCL12 at 0, 0.003, 0.03, or 0.3 nM concentrations. Data was normalized to control siRNA transfected, vehicle-treated cells. *ANOVA* was used to determine significant differences (*p ≤ 0.05, n = 3) between control and experimental cells. (C-E) Western blots to test the effects of CXCR7 and CXCR4 knockdown on AR signaling in LNCaP cells. Cells were transfected with control, CXCR7 or CXCR4 siRNA for 72 hrs and probed with antibodies against **(C)** CXCR7, **(D)** CXCR4, **(E)** AR, and PSA. The densitometry values were normalized to control siRNA transfected cells and labeled below the blots. **(F)** Silver staining demonstrates equivalent loading across the samples.

Given that CXCL12 can stimulate or repress cell motility in a dose-dependent manner in monocytic leukemia and colon cancer cell lines [18,50], we wanted to determine the CXCL12 concentration that is optimal for stimulating CXCR4-mediated motility in LNCaP cells. All CXCL12-dependent cell motility assays were performed in the presence of 1 nM androgen, the dose that promoted maximal motility (Figure 7A). A bare filter cell migration assay was performed on LNCaP cells treated with control (0.1% BSA) or various concentrations of CXCL12 (*i.e.,* 0.3 nM, 3 nM, and 30 nM) (Figure 8A). As shown in Figure 8A, CXCL12 induced a biphasic cell motility response in LNCaP cells, with maximum cell motility observed at 0.3 nM CXCL12. Moreover, the increase in cell motility was not due to an increase in cell proliferation under the conditions of the cell motility assay (Additional file 2: Figure S2.C). These results demonstrate that CXCL12 mediates a dose-dependent cell motility response in LNCaP cells.

Previous studies have shown that a reduction in CXCR7 expression enhances chemotaxis in response to low levels of CXCL12 in human T-cells, yet in the migrating neurons of mice, Cxcr7 prevents Cxcl12-mediated desensitization of Cxcr4 due to stabilization of Cxcr4 protein [55,56]. Therefore, we wanted to determine if the expression of CXCR4 or CXCR7 had any influence on the biphasic CXCL12-mediated cell motility response in LNCaP cells. Migration assays were performed on LNCaP cells transfected with control, CXCR4, or CXCR7 siRNAs and treated with control (0.1%BSA) or various concentrations of CXCL12 (0.003 nM, 0.03 nM, and 0.3 nM) (Figure 8B). As shown in Figure 8B, the maximal motility response in control cells was observed at 0.3 nM CXCL12, whereas maximal motility in the CXCR7 knockdown cells was observed at 0.03 nM CXCL12. This result showed that the maximum response was shifted to a 10-fold lower concentration in the CXCR7 knockdown cells (Figure 8B). In contrast, the cell motility response was severely blunted in CXCR4 knockdown cells (Figure 8B), highlighting the functional significance of CXCR4 signaling in CXCL12-mediated chemotaxis.

Next, we examined co-dependent gene expression relationships between CXCR7 and CXCR4, since in breast cancer cells CXCR7 is known to regulate CXCL12-mediated cell motility by modulating CXCR4 expression [37]. Whole-cell lysates isolated from LNCaP cells transfected with control, CXCR7, or CXCR4 siRNAs for 96 hrs were subjected to western blot analysis to quantify CXCR7 and CXCR4 levels (Figure 8C and D). Higher levels of CXCR7 were detected in CXCR4 knockdown cells relative to controls (Figure 8C, compare lane 3 to lane 1), suggesting that CXCR4 expression attenuated CXCR7 levels in LNCaP cells. In contrast, lower levels of CXCR4 were detected in CXCR7 and CXCR4 knockdown relative to control cells. (Figure 8D, compare lane 2 and 3 to lane 1). Moreover, AR and PSA levels were reduced in CXCR7 knockdown relative to control cells (Figure 8E, compare lane 2 to lane 1) but were relatively unchanged in CXCR4 knockdown cells (Figure 8E, compare lane 3 to lane 1). Overall, these results show that homeostatic levels of CXCR4 protein require CXCR7 expression, and that CXCR4 expression is required for CXCL12-mediated motility in LNCaP prostate-cancer cells.

## Discussion

Androgens have been shown to increase the metastatic potential of prostate tumor cells by upregulating the expression of CXCR4 [8,25]. In cells positive for the TMPRS22-ERG gene fusion, androgens promote CXCR4 expression through the transcriptional actions of the oncogenic ETS-family transcription factor ERG; in TMPRS22-ERG negative cells, it occurs through the transcriptional upregulation of the KLF5 transcription factor [25]. In the context of human prostate cancer development, CXCR4 expression is higher in localized prostate-cancer cells than in the surrounding normal tissue (Table 1). This suggests that CXCR4 expression is antagonized under homeostatic conditions and in the presence of circulating androgens, but that this suppression is relieved in hormone-naïve organ-confined prostate tumor cells. CXCR7 expression, on the other hand, is lower in localized prostate cancers than normal prostate tissue, suggesting that homeostatic, physiological levels of androgens enhance CXCR7 expression (Table 1). Overall, gene expression analyses of clinical samples show that androgenic control of the CXCR4/CXCR7 axis becomes corrupted when normal prostate epithelial cells are transformed into organ-confined prostate-cancer cells [32-35]. This corrupted signaling continues in sex-steroid-responsive prostate and breast tumor-cell lines [29,30]. In established cellular models of human prostate and breast cancer, androgens and estrogens engage the CXCR4/CXCR7 axis by stimulating or inhibiting CXCR4 and CXCR7 expression, respectively [25,57,58]. In this study we showed that the synthetic androgen R1881 reciprocally regulates CXCR4 and CXCR7 expression at the protein level in androgen-sensitive human LNCaP prostate tumor cells. In these cells, the *in vitro* cell-motility response to androgen was dose-dependent and biphasic, with maximal motility observed at physiological levels of androgen (*e.g.,* 1 nM R1881) (Figure 7A). Previous studies had shown that the proliferation of hormone-sensitive prostate tumor cells (*e.g.,* LNCaP cells) is biphasic and dependent on the dose of androgen, and maximal proliferation occurs at physiological concentrations (*i.e.,* 1 nM R1881) [59-61]. Notably, we found that a supraphysiologic concentration of androgen (*i.e.,* 10 nM R1881) antagonized the motility of LNCaP cells; this same treatment is also known to antagonize their proliferation *in vitro* [59-61]. It will be interesting to explore if supraphysiologic levels of androgen antagonize cell motility and proliferation *in vivo*, since these cellular processes are determinants of the tumorigenic potential of human prostate-cancer cells.

We have also provided indirect biochemical evidence that membrane-localized CXCR4 and CXCR7 are potentially glycosylated (*e.g.,* changes in N- and O-linked glycosylation) in response to androgens in LNCaP cells (Figure 7B). Although CXCR4 is known to harbor N-linked glycans [62], future experiments will be required to unequivocally determine if CXCR7 is also N-linked glycosylated in prostate-cancer cells. Androgens may also induce other post-translational modifications on CXCR4 and CXCR7 (*e.g.*, ubiquitination or phosphorylation) [63,64]. Androgens are known to increase the cell surface expression of CXCR4 in LNCaP cells [25], and our data extend these findings to show that androgens also influence the intracellular expression and colocalization of CXCR4 and CXCR7 in LNCaP cells (Figure 7F). The optimal dose of synthetic androgen with respect to promoting androgen-mediated motility correlated with the maximal intracellular co-localization of CXCR4 and CXCR7 in LNCaP cells (Figure 7A and F). These results suggest that androgen-mediated cell motility occurs in response to physical interactions between CXCR4 and CXCR7 in LNCaP cells. Additionally, CXCR7 knockdown attenuated the motility of LNCaP cells in response to CXCL12 (Figure 8B), a result consistent with a previous demonstration that the dose–response curve to CXCL12 shifted downward in CXCR7 knockdown cells, allowing T-cell chemotaxis at lower CXCL2 concentrations [18]. Recent mouse studies have shown that CXCR7 prevented CXCL12-mediated desensitization of CXCR4 in migrating interneurons [55]. The mechanisms by which CXCR7 does so remain an active topic of investigation. Multiple studies suggest that it either acts as a scavenger, sequestering CXCL12, or attenuates CXCR4 signaling by forming CXCR7/CXCR4 heterodimers that influence CXCR4 stability and degradation, possibly through interactions with β-arrestin2 [17,18,49,65,66]. Both models could explain why CXCL12-mediated cell motility was extinguished in CXCR7 knockdown cells at lower concentrations of CXCL12 (Figure 8B).

CXCR7 expression was also required for CXCR4 homeostasis in LNCaP prostate-cancer cells, as CXCR4 protein levels were substantially reduced in the context of CXCR7 knockdown (Figure 8D) [55]. CXCR7 levels were increased in CXCR4 knockdown cells, demonstrating that CXCR4 antagonizes CXCR7 protein levels in LNCaP cells (Figure 8C). Thus, CXCR7 and CXCR4 are reciprocally regulated in LNCaP cells, with CXCR7 promoting CXCR4 expression, but CXCR4 antagonizing CXCR7 expression. To the exclusion of transcription-based mechanisms for regulating CXCR7 expression by CXCR4, other mechanisms including protein degradation may also contribute to CXCR4-based antagonism of CXCR7 expression. More importantly, an increase in CXCL12-mediated motility was not observed in CXCR4 knockdown cells despite an increase in CXCR7 protein (Figure 8B and C). These results provide further support for the idea that CXCL12-mediated cell motility is CXCR4-dependent in LNCaP cells, and that CXCR7 expression alone is insufficient to explain CXCL12-mediated cellular motility. Additionally, our data in LNCaP cells support the current cellular model where the primary role of CXCR7 during CXCL12-mediated cell motility is to prevent CXCR4 desensitization by scavenging CXCL12 [17,37,55]. A recent study also showed that CXCR7-positive tumor cells promoted the metastasis of CXCR4-positive breast tumor cells [37]. Metastasis did not require cell-autonomous co-expression of both receptors; instead, the co-implantation of CXCR7- and CXCR4-positive tumor cells was sufficient to promote the metastasis of the latter, and this involved scavenging of extracellular CXCL12 [37]. Moreover, our study shows that CXCR7 regulates CXCR4 levels and is required for optimal CXCL12-dependent motility at the 0.3 nM concentration of CXCL12 (Figure 8B and D). Overall, our findings are consistent with CXCR7 modulating CXCL12-mediated motility by regulating CXCR4 expression [17,37,55,56].

**Figure 9:**
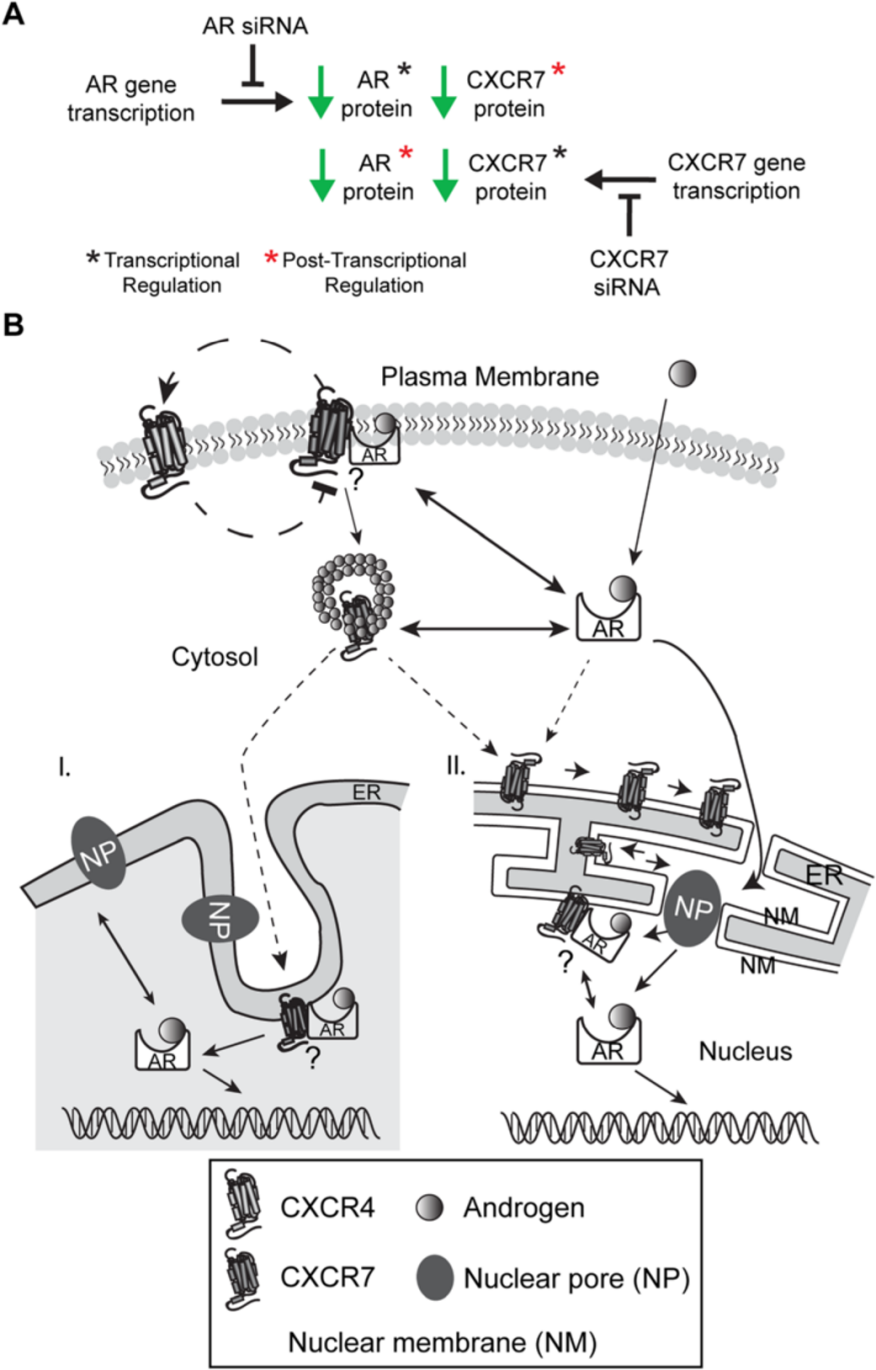
**Speculative models for CXCR7 signaling in prostate cancer cells. (A)** CXCR7 and AR protein levels are modulated by AR and CXCR7 respectively, through post-transcriptional mechanisms (*e.g.,* protein stability). **(B)** Reciprocal feedback loop regulating the expression of CXCR4 and CXCR7 in LNCaP prostate cancer cells, including protein trafficking pathways that could account for the transport of CXCR7 into the nuclear compartment. Question mark indicates that AR may directly or indirectly interact with CXCR7. (I) CXCR7 gains access to the nucleus through the nucleoplasmic reticulum (*e.g.* invaginations of the nuclear envelope) [73,77]. (II) CXCR7 gains access to the nucleus through the nuclear pore complex (*e.g.,* transportin-dependent), as shown for other GPCRs and more recently for CXCR4 [69,70].

An unexpected finding of our study was that CXCR7 was present in the nucleus, in both androgen-sensitive (*i.e.,* LNCaP, 22Rv1) and androgen refractory (*i.e.,* PC-3, DU145) human prostate-tumor cells (Figure 2B, and Additional file 1: Figure S1.D). A growing number of studies have reported the nuclear localization of classical GPCRs in human cell lines and tissues (*e.g.,* gonadotropin releasing hormone receptor-GnRH-R, beta-adrenergic receptors-β_1_ARs and β_3_ARs) [67-69]. Notably, CXCR4 was shown to undergo transportin 1-dependent nuclear localization in cancerous prostate tissues and established, metastatic prostate-cancer cell lines [70]. Moreover, CXCR4 isolated from the nuclei of PC-3 cells was capable of mediating G-protein signaling in response to CXCL12 [70]. We carried out a bioinformatics analysis of the primary sequence of CXCR4, using PSORT II, and uncovered a putative nuclear localization sequence (NLS) (*e.g.,* RPRK) that contributed to CXCR4 nuclear localization. However, this NLS does not fall within the primary sequence of CXCR7 (Additional file 3: Table S1), suggesting that the nuclear localization may involve a transportin 1-independent mechanism(s). For example, CXCR7 might gain access to the nucleus through the nucleoplasmic reticulum, which brings the endoplasmic reticulum and chromatin into close proximity and can influence nuclear calcium signaling [71-73]. A model for how a hydrophobic 7-transmembrane receptor such as CXCR7 might traffic to the nucleus to modulate gene transcription is shown in Figure 9B. Future studies are warranted to test the validity of such mechanisms in prostate-cancer cells and to determine whether these are features that are restricted to neoplastic prostate epithelial cells.

Unexpectedly, we observed a physical interaction between CXCR7-SBP and AR in C7-SBP cells, and functional interactions between CXCR7 and AR in LNCaP prostate-cancer cells (Figures 4, 5, and 6). Based upon the co-localization of AR and CXCR7 proteins it is highly likely AR, CXCR7, and β-arrestin 2 form a ternary complex since β-arrestin 2 has been shown to bind both AR and CXCR7 in prostate-cancer cells [19,46,74,75]. Further biochemical experiments will delineate whether AR and CXCR7 interact directly or indirectly in prostate-cancer cells. AR-mediated transcription was modulated by changes in CXCR7 expression, demonstrating crosstalk between the CXCR7 and AR signaling pathways (Figure 6). Most interestingly, CXCR7/AR knockdown had an addictive effect on AR, CXCR7 and PSA levels (Figure 4A). This additive interaction suggests that AR and CXCR7 act on separate unrelated processes to reciprocally regulate their protein expression in LNCaP prostate-cancer cells [76]. Given the increased expression of the AR mRNA in CXCR7 knockdown cells (Figure 1B), CXCR7 most likely modulates AR protein levels through post-transcriptional mechanisms in LNCaP cells (Figure 9A). Conversely, CXCR7 protein levels are likely modulated post-transcriptionally by AR. We propose a model where both proteins act to reciprocally regulate their levels in LNCaP cells (Figure 9A). Future studies will seek to elucidate this post-transcriptional relationship at the molecular level. Based upon the physical interaction of CXCR7-SBP with AR in C7-SBP cells, we speculate that AR and CXCR7 protein levels might be regulated through protein-protein interactions mediated between AR and CXCR7 in androgen-sensitive prostate-cancer cells. Notably, crosstalk between the CXCL12/CXCR4 axis and the AR signaling pathway in established prostate-cancer cell lines (*i.e.,* LNCaP, 22Rv1 cells) was reported previously [51], with chronic CXCL12 stimulation inducing: nuclear translocation of AR; the transcription of androgen-regulated genes (*e.g., PSA*, *TMPRSS2*); the association of AR with known AR co-regulators (*e.g.,* SRC-1); and cell proliferation in the context of serum-free growth conditions [51]. Nevertheless, biochemical evidence establishing a molecular link between the CXCL12/CXCR4 axis and the AR signaling pathway was not provided. Our results do so for LNCaP prostate-cancer cells (Figures 4, 5, and 6). Surprisingly, pre-treatment of *AD*-LNCaP cells with CXCL11 or CXCL12 disrupted androgen-mediated expression of the *probasin*-luciferase vector. More importantly, CXCL11 and CXCL12 were able to attenuate the expression of endogenous *ARGs*, demonstrating crosstalk between chemokine/chemoreceptor pathways and AR-mediated gene expression programs in prostate-cancer cells (Figure 6C). In light of the finding that CXCR7 expression is required for normal AR transcriptional activity, the pre-engagement of CXCL11 or CXCL12 with CXCR7 in the context of androgen depletion likely disrupts the physical interactions between CXCR7 and AR that promote maximal AR transcriptional activity in prostate-cancer cells. Future studies are warranted to unravel the mechanism(s) by which chemokines CXCL11 and CXCL12 engage the AR signaling program at the molecular level. This information may allow us to manipulate chemokine pathways to disrupt the aberrant androgen-mediated cellular processes (*i.e.*, cellular proliferation, cell motility) that contribute to the progression of human prostate cancers.

## Conclusions

In summary, our findings in androgen-sensitive prostate tumor cells reveal that androgens modulate cell motility in a dose-dependent manner in response to chemokine CXCL12, and that they do so by regulating the expression of chemokine receptors CXCR4 and CXCR7. In addition, biochemical and co-localization data support a physical interaction between CXCR7 and AR, in establishing a molecular link between the chemokine-dependent signaling axis and AR signaling pathways. Further molecular dissection of how AR modulates the CXCL11/CXCL12/CXCR4/CXCR7 axis to influence the motility of prostate tumor cells is expected to facilitate the development of new hormone-based strategies that are capable of decreasing the metastatic potential of localized prostate cancers exposed to androgens.

## Additional files

Additional file 1: Figure S1. CXCR7 expression in prostate-cancer cells. (A) Western blot of whole cell lysates from LNCaP cells transfected with control or CXCR7 siRNA (100 nM) for 72 hrs with Ab72100 or Ab38089 antibodies. Ab38089 antibody only recognized the 60-kDa immunoreactive band. The densitometry values were normalized to control siRNA transfected cells and labeled below the blots. (B) Western blot analysis of LNCaP cells stably expressing SBP-tag or CXCR7 with a SBP-tag on the C-terminus (C7-SBP) using the pAbCXCR7 and SBP antibodies. (C) Western blot of lysates from multiple human cell lines with the pAbCXCR7 antibody. Sample extracts were resolved into a 12% SDS polyacrylamide gel. (D) Immunofluorescence analysis of 22Rv1 (I-I to I-III), DU145 (II-I to II-III), and PC3 (III-I to III-III) prostate-cancer cells with antibodies against CXCR7 and treated with DAPI.
Additional file 2: Figure S2. (A) Quantitation of 1,000, 2,000, 3,000, 4,000, 5,000, and 8,000 LNCaP cells using the CyQuant Cell Proliferation Assay Kit. (B-C) Quantitative measurement of LNCaP cells grown in different doses of (B) androgen (R1881), or (C) CXCL12-treated cells for 24 hrs. *ANOVA* was used to determine significant differences (*p ≤ 0.05, n = 3) between samples, and no statistical significant differences were found.
Additional file 3: Table S1. The human CXCR4 (UniProt ID: P61073) and CXCR7 (UniProt ID: P25106) sequences were searched using PSORT II, and the predicted NLS sequence for CXCR4 is shown.

## Abbreviations

*AD*: Androgen-depleted
AR: Androgen receptor
CXCL11: Chemokine ligand 11
CXCL12: Chemokine ligand 12
CXCR4: C-X-C chemokine receptor type 4
CXCR7: C-X-C chemokine receptor type 7
PSA: Prostate specific antigen
SBP: Streptavidin binding peptide
TMPRSS2: Transmembrane protease, serine 2
