# Supplementary figures and images for: Androgen receptor and chemokine receptors 4 and 7 form a signaling axis to regulate CXCL12-dependent cellular motility
Source: BMC Cancer. 2015 Mar 31;15:204. doi: 10.1186/s12885-015-1201-5 (PMC4393632; doi:10.1186/s12885-015-1201-5)

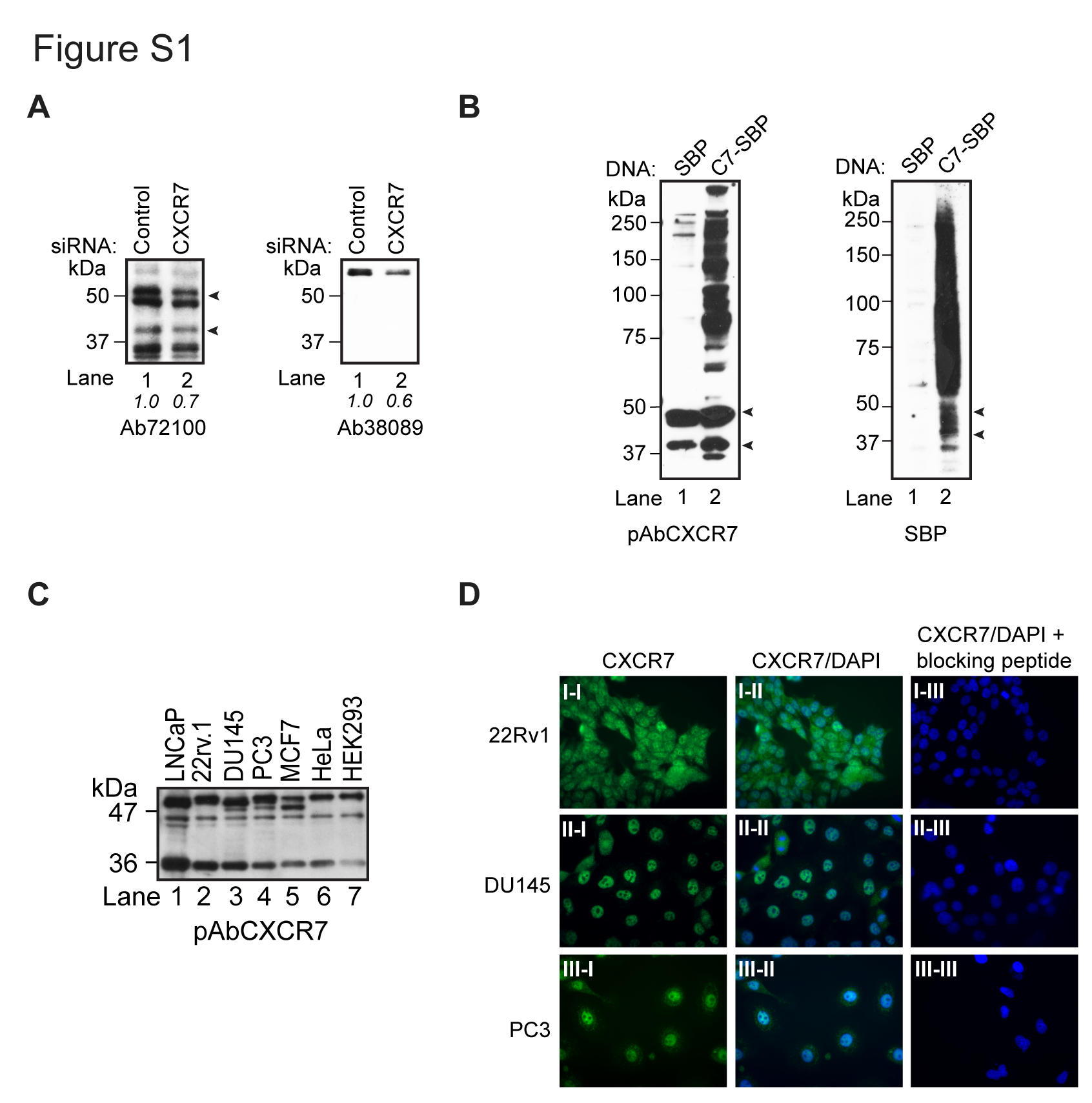

Supplement: Additional file 1: Figure S1. — CXCR7 expression in prostate-cancer cells. (A) Western blot of whole cell lysates from LNCaP cells transfected with control or CXCR7 siRNA (100 nM) for 72 hrs with Ab72100 or Ab38089 antibodies. Ab38089 antibody only recognized the 60-kDa immunoreactive band. The densitometry values were normalized to control siRNA transfected cells and labeled below the blots. (B) Western blot analysis of LNCaP cells stably expressing SBP-tag or CXCR7 with a SBP-tag on the C-terminus (C7-SBP) using the pAbCXCR7 and SBP antibodies. (C) Western blot of lysates from multiple human cell lines with the pAbCXCR7 antibody. Sample extracts were resolved into a 12% SDS polyacrylamide gel. (D) Immunofluorescence analysis of 22Rv1 (I-I to I-III), DU145 (II-I to II-III), and PC3 (III-I to III-III) prostate-cancer cells with antibodies against CXCR7 and treated with DAPI. [file 12885_2015_1201_MOESM1_ESM.tiff]

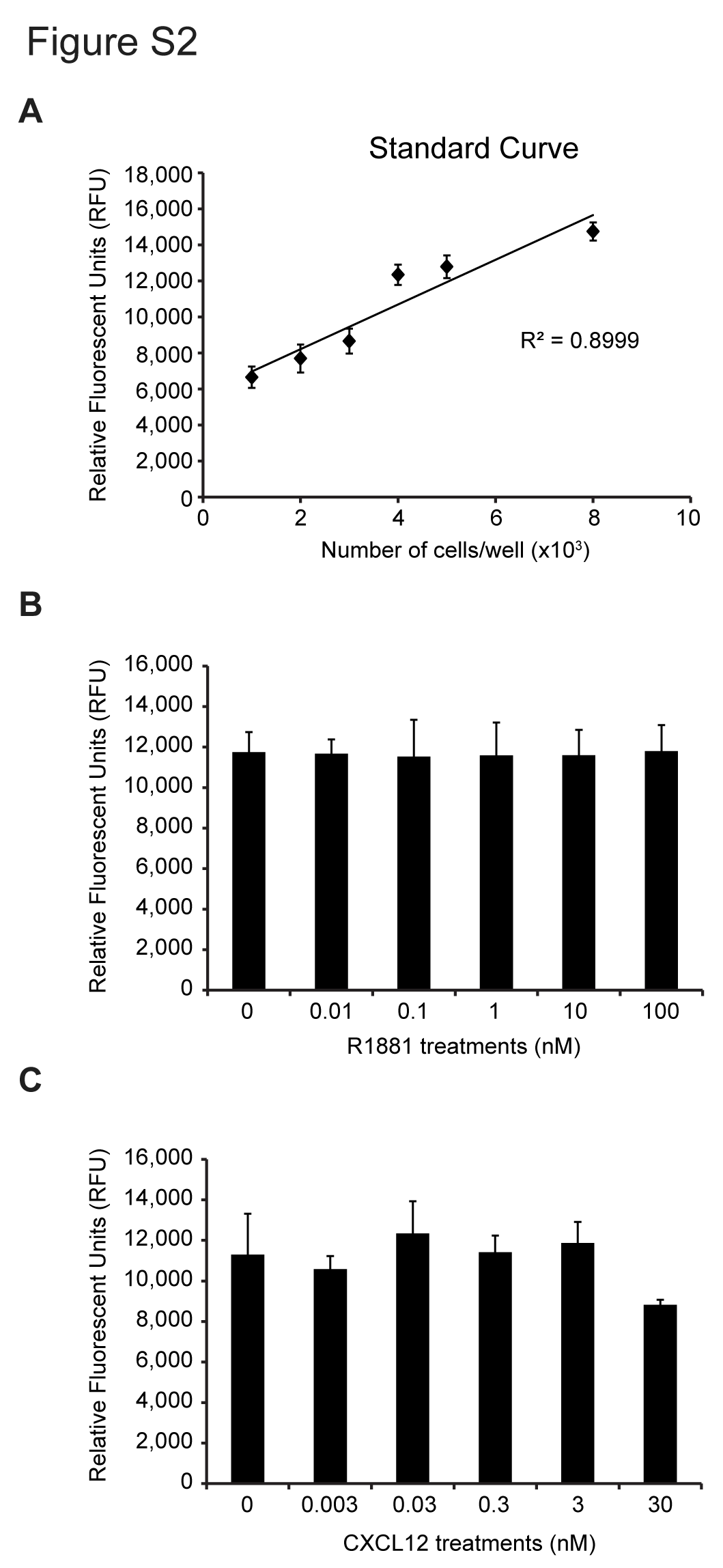

Supplement: Additional file 2: Figure S2. — (A) Quantitation of 1,000, 2,000, 3,000, 4,000, 5,000, and 8,000 LNCaP cells using the CyQuant Cell Proliferation Assay Kit. (B-C) Quantitative measurement of LNCaP cells grown in different doses of (B) androgen (R1881), or (C) CXCL12-treated cells for 24 hrs. ANOVA was used to determine significant differences (*p ≤ 0.05, n = 3) between samples, and no statistical significant differences were found. [file 12885_2015_1201_MOESM2_ESM.tiff]

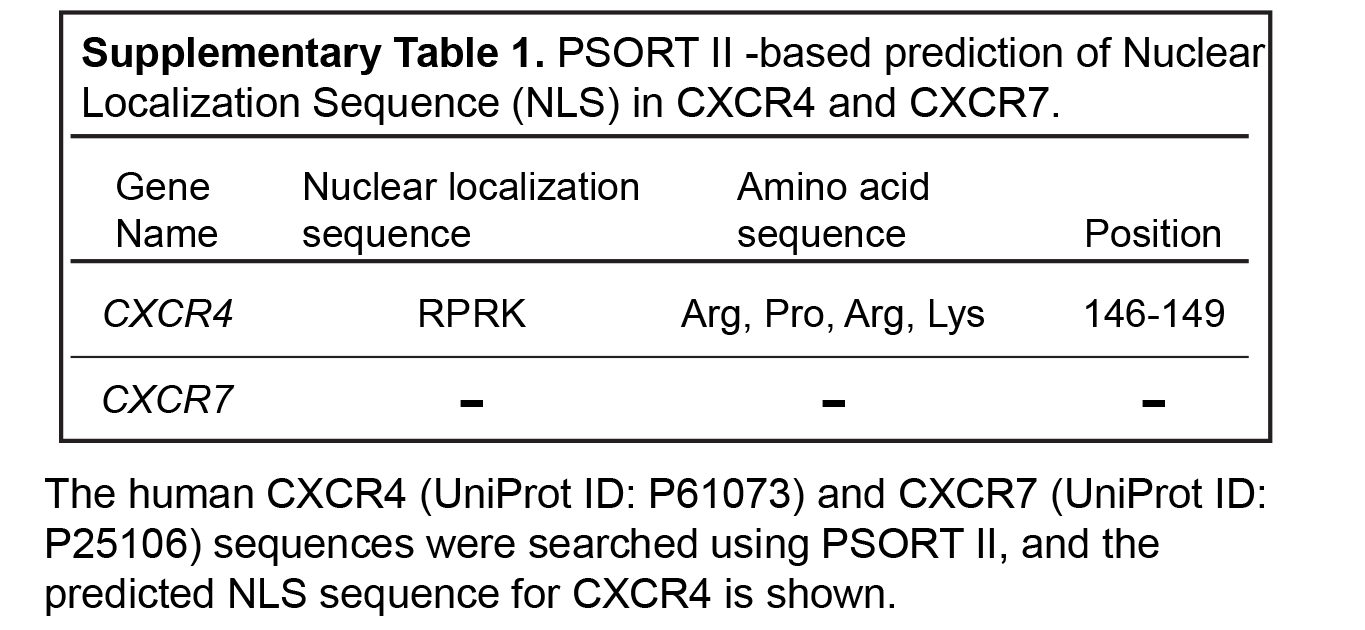

Supplement: Additional file 3: Table S1. — The human CXCR4 (UniProt ID: P61073) and CXCR7 (UniProt ID: P25106) sequences were searched using PSORT II, and the predicted NLS sequence for CXCR4 is shown. [file 12885_2015_1201_MOESM3_ESM.tiff]
